# Postoperative Risk and Climate Exposure: A Retrospective Study of Incisional Glaucoma Surgery Outcomes

**DOI:** 10.1167/tvst.15.4.24

**Published:** 2026-04-27

**Authors:** Tianhao Chen, Yi Tian, Yixiang Zhu, Jiaying Li, Xinghuai Sun, Haidong Kan, Yuyan Zhang, Yuan Lei

**Affiliations:** 1 Eye Institute and Department of Ophthalmology, Eye & ENT Hospital, Fudan University, Shanghai, China; 2 Shanghai Medical College, Fudan University, Shanghai, China; 3 School of Public Health, Key Lab of Public Health Safety of the Ministry of Education, NHC Key Lab of Health Technology Assessment, IRDR ICoE on Risk Interconnectivity and Governance on Weather/Climate Extremes Impact and Public Health, Fudan University, Shanghai, China; 4 Department of Ophthalmology, Longhua Hospital, Shanghai University of Traditional Chinese Medicine, Shanghai, China; 5 NHC Key laboratory of Myopia and Related Eye Diseases; Key Laboratory of Myopia and Related Eye Diseases, Chinese Academy of Medical Sciences, Shanghai, China; 6 Shanghai Key Laboratory of Visual Impairment and Restoration, Shanghai, China; 7 State Key Laboratory of Medical Neurobiology and MOE Frontiers Center for Brain Science, Institutes of Brain Science, Fudan University, Shanghai, China; 8 Children's Hospital of Fudan University, National Center for Children's Health, Shanghai, China

**Keywords:** incisional glaucoma surgery, ambient temperature, U-shaped association, air pollution, interaction

## Abstract

**Purpose:**

To evaluate associations between ambient temperature and surgical failure after incisional glaucoma surgery.

**Methods:**

Individual-level clinical data were obtained from electronic medical records. Ambient temperature was estimated via spatiotemporal bilinear interpolation. Multivariable random-effects Cox proportional hazards models, incorporating restricted cubic splines and segmented regression, assessed the temperature-outcome relationship. Stratified analyses identified potentially susceptible populations. Likelihood ratio tests examined interactive effects between air pollution and ambient temperature on both multiplicative and additive scales.

**Results:**

Although conventional linear analyses did not show a significant association between ambient temperature and surgical failure risk, a spline-based nonlinear models suggested a U-shaped, asymmetric nonlinear association between ambient temperature and surgical failure risk within prolonged exposure windows (*P*_nonlinearity < 0.0001). Under these nonlinear specifications, exposure to temperature extremes relative to the mid-range was associated with an increased estimated risk of surgical failure. These nonlinear patterns appeared more evident in older patients and those with primary open-angle glaucoma. Air pollutants showed statistically significant effect modification in interaction analyses of the temperature–outcome association 180 days after surgery. With the exception of O₃, common air pollutants demonstrated negative interaction terms with temperature, suggesting that the modeled association between lower temperatures and surgical failure risk was stronger at higher pollutant concentrations.

**Conclusions:**

Nonlinear modeling suggested potential heterogeneity in risk across temperature ranges, with the estimated associations varying by PM₂.₅ exposure strata and other air pollutant levels. These findings are model-dependent and warrant confirmation in independent cohorts and prospective studies.

**Translational Relevance:**

Under spline-based nonlinear modeling, postoperative ambient temperature showed a U-shaped association with glaucoma surgical failure risk, with the estimated relationship differing across levels of concurrent air pollutant exposure. These findings are hypothesis-generating and suggest that environmental conditions may warrant consideration in postoperative risk assessment.

## Introduction

Glaucoma remains the leading cause of irreversible blindness worldwide, and surgical intervention is often required when medical and laser treatments fail to provide adequate intraocular pressure (IOP) control.^1^^,^^2^ Although clinically effective, these surgical procedures are frequently hindered by postoperative conjunctival fibrosis and bleb failure, which compromise long-term success.^3^ Identifying modifiable risk factors that contribute to surgical failure is essential for improving visual prognosis in patients with advanced disease.

Emerging evidence suggests that ambient temperature may influence IOP homeostasis, a key determinant of both disease progression and surgical outcomes. Cold exposure elevates IOP by reducing aqueous humor outflow^4^ and increasing fluid viscosity.^5^ Conversely, thermal stress may trigger inflammation,^6^ fibroblast proliferation, and extracellular matrix deposition^7^—key drivers of postoperative scarring. Although several epidemiologic studies have linked ambient temperature with glaucoma incidence and progression,^8^^,^^9^ its impact on surgical outcomes remains insufficiently explored.

To address these knowledge gaps, we conducted a retrospective cohort study to evaluate the association between ambient temperature and outcomes after incisional glaucoma surgery. Given established evidence that air pollution modulates temperature-related health effects,^10^^–^^15^ we further investigated its potential modifying role and interaction with temperature regarding surgical outcomes. Against the backdrop of escalating extreme weather events driven by climate change and growing emphasis on regulating pollutant levels, consideration of these modifiable risk factors may contribute to our insight into the association between environmental factors and surgical outcomes.

## Methods

### Study Population

**Figure 1.**
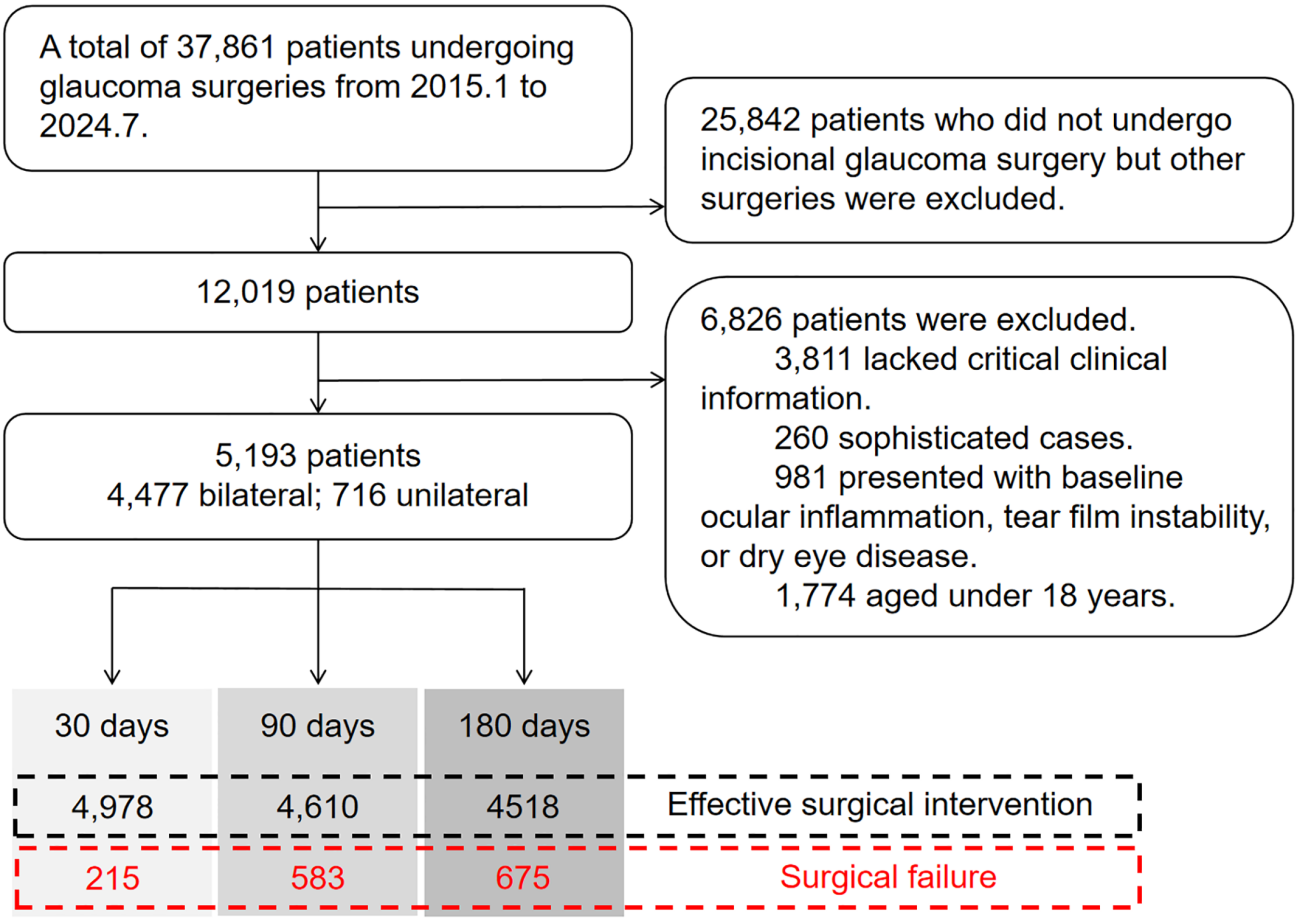
The flow chart of the enrolled participants.

We identified 31,973 Chinese patients who underwent incisional glaucoma surgery between January 2015 and July 2024 across two clinical databases from the database of Eye and Ear, Nose, Throat Hospital of Fudan University and Huadong Hospital Affiliated to Fudan University. Of these, 25,842 patients were excluded for undergoing non-incisional glaucoma procedures. Further exclusions were made for patients who lacked critical information (including residential address, postoperative visit and medication use) and exposure data, or presented with ocular inflammation, tear film instability or dry eye disease. We additionally excluded complex cases undergoing another glaucoma surgery before the visit to hospitals we collect information and those exposed to extreme temperatures (outside the 2.5th to 97.5th location-specific percentiles) to minimize selection bias.^16^ In total, 5193 patients aged over 18 years with complete demographic and clinical data were selected (Fig. 1). This cohort comprised 716 patients who underwent bilateral surgery and 4477 who received unilateral surgery. The study protocol was approved by the Institutional Review Board of the Eye and ENT Hospital of Fudan University (IRB no. 2024 184) and adhered to the tenets of the Declaration of Helsinki. Given the study's retrospective nature, the requirement for informed consent was waived.

### Exposure Data Collection and Analysis

The longitude and latitude coordinates of each patient's residence were retrieved from the hospital database, and no known migration occurred during targeted period. Temperature and relative humidity exposure data at 0.1° × 0.1° horizontal resolution were obtained from ERA5-LAND data set (https://cds.climate.copernicus.eu/datasets/reanalysis-era5-land?tab=overview).^17^ Exposure data of PM_2.5_ and PM_10_ were derived from ChinaHighAirPollutants (http://data.tpdc.ac.cn).^18^^–^^22^ It is generated from big data (including ground-based measurements, satellite remote sensing products, atmospheric reanalysis, and model simulations) using artificial intelligence by considering the spatiotemporal heterogeneity of air pollution. This dataset yields a high quality for both PM_2.5_ and PM_10_, with a cross-validation coefficient of determination (CV-R^2^) of 0.90 and 0.92, a root-mean-square error (RMSE) of 21.12 µg/m^3^ and 10.76 µg/m^3^, and a mean absolute error of 11.22 µg/m^3^ and 6.32 µg/m^3^ on a daily basis, respectively. We used bilinear interpolation to estimate the levels of ambient temperature subsequent to glaucoma surgery at patients’ resident addresses.^23^ Other pollutant (NO_2_, SO_2_, O_3_, and CO) exposure data were obtained from retrieved in China's National Urban Air Quality Real-time Publishing Platform. The analyses included daily 24-hour average levels of temperature and relative humidity, and daily 24-hour average levels of PM_2.5_, PM_10_, SO_2_, NO_2_ and CO, as well as daily eight-hour maximum average levels of O_3_ to investigate the effect of air pollution in temperature-induced impact on surgical outcomes.

### Definition of Surgical Failure

Surgical failure was defined according to previous studies^24^^,^^25^ and established clinical guidelines^26^ and was considered to have occurred if either of the following criteria was met: (1) Failure of IOP control: IOP > 21 mm Hg on two consecutive postoperative assessments; or a reduction in IOP of less than 20% from preoperative baseline levels, irrespective of the use of IOP-lowering medications; (2) Need for re-intervention: a second glaucoma surgery or surgical revision performed due to bleb dysfunction, inadequate IOP control, or related complications. Given the reported characteristics of wound healing and fibrotic response following filtration surgery in Asian populations, we evaluated the earlier risk of surgical failure occurring within one, three, and six months after surgery to assess the association between ambient temperature and early surgical failure.

### Covariates

The covariates included age, sex, economic geographical location (east, east-north, middle or west), baseline IOP, preoperative ophthalmic solutions usage (prostaglandin analogs, β-blockers, α2-adrenergic agonists, carbonic anhydrase inhibitors, cholinergic agents, fixed-combination drugs, anti-inflammatory drugs, unknown medications, or no medications; Supplementary Table S1) and duration, surgery season, surgery type (trabeculectomy, Ex-PRESS shunt or Ahmed valve), systematic comorbidities (hypertension and diabetes) and glaucoma subtype (primary open-angle glaucoma [POAG], primary angle-closure glaucoma [PACG], and secondary glaucoma).^27^^–^^29^ Additionally, intraoperative and postoperative antifibrotic agent (5-fluorouracil [250 mg per vial, box of five vials], mitomycin C [MMC; 10 mg per box] or no agent use) was included as another covariate.^30^

### Surgical Procedure and Antifibrotic Treatment

As a multicenter study, inherent variations in surgical techniques across centers reflect real-world clinical practice. To ensure the consistency and comparability of the core procedure—antifibrotic agent application—we examined standardized protocols from two hospitals focusing on key steps: the concentration and exposure time of MMC/5-FU, the precise placement of sponges in the subconjunctival and sub-Tenon's space, and mandatory thorough irrigation.^30^ For patients receiving intraoperative antifibrotics, 5 × 10 mm sponges soaked in MMC or 5-FU were placed in the subconjunctival and sub-Tenon spaces at the surgical site. The sponges remained in place for two to three minutes. After the exposure period, the sponges were carefully removed, and the treated area was thoroughly irrigated with balanced salt solution or sterile saline solution to minimize MMC toxicity and prevent residual effects.

### Statistical Analysis

#### Data Reporting

Continuous variables were reported as mean ± standard deviation or median (interquartile range), while categorical variables were summarized as numbers and percentages. The *t*-test or analysis of variance was applied for continuous variables following a normal distribution, and the Mann-Whitney U test or Kruskal-Wallis test was used for non-normally distributed variables. Pearson's χ^2^ test was used to compare categorical outcomes.

#### Survival Analyses

Survival time was defined as the interval from surgery to the onset of surgical failure or the exposure window we set manually (30, 90, or 180 days). We deployed Kaplan-Meier survival analysis to ascertain different incidence rates within groups defined by ambient temperature exposure level, with log-rank test determining the statistical significance.

#### Random-Effects Cox Proportional Hazards Regression

To appropriately account for the within-patient correlation in bilateral eye surgery outcomes, we used a random-effects Cox proportional hazards model with patient-level frailty terms. This approach allows for the simultaneous estimation of fixed covariate effects and random individual heterogeneity while maintaining the proportional hazards assumption. The hazard function for the *i*th patient's *j*th eye was defined as

$$\begin{array}{c} \lambda_{ij}\left(t\right)\;=\;\lambda_{0}\left(t\right)exp\left(\beta X_{ij}\;+\;\mu_{i}\right) \end{array}$$

where µ_*i*_ represents the normally distributed random effect for each patient, *X_ij_* denotes all covariates (e.g., surgical factors, demographics), and λ_0_(*t*) is the nonparametric baseline hazard. The results were presented as the hazard ratios (HRs) of surgical failure incidence associated 1°C with the 95% confidence intervals (CI) of ambient temperature.

#### Restricted Cubic Splines and Threshold Effect Analysis

Given that environmental exposures often exhibit nonlinear relationships with health outcomes, we pre-specified the use of restricted cubic splines (RCS) to explore potential nonlinear associations between temperature and surgical failure risk, building upon our primary linear/categorical models. The curve was fitted with a pre-specified four-knot placement (at the fifth, thirty-fifth, sixty-fifth, and ninety-fifth percentiles), and robustness was further verified by varying the number of knots. Additionally, we constructed threshold effect analysis to assess the relative low-risk ranges. Methodologically, to enhance precision, we applied maximum likelihood estimation to determine temperature inflection points and validated findings using bootstrap resampling to establish CIs.^31^

To compare the nonlinear model against a linear specification, we performed a likelihood ratio test. The likelihood ratio test statistic was calculated as twice the difference in log-partial-likelihood between the RCS model and the nested linear Cox model, which was then compared to a χ^2^ distribution with degrees of freedom equal to the difference in the number of parameters (corresponding to the nonlinear spline terms). Further model selection evidence was provided by comparing the Akaike information criterion (AIC) and Bayesian information criterion (BIC) between the two specifications. To assess potential overfitting and the internal validity of the RCS model, we performed bootstrap validation (1000 resamples) to estimate the optimism in the model's concordance index (C-index).

#### Stratification and Interaction Analyses

Stratification and interaction analyses were conducted to assess the influence of sex, age (<60 years vs. ≥60 years), baseline IOP, glaucoma subtype, intraoperative drug, and surgery type on the relationship between ambient temperature and surgical failure. Likelihood ratio tests were used to evaluate potential interactions. Additionally, to evaluate the interaction effect between air pollution and ambient temperature on glaucoma surgical outcomes, we first tested the modification on a multiplicative scale (using HR for the product term between specific air pollutant and ambient temperature) using the likelihood ratio test. Second, after dichotomizing air pollutant levels, additive interaction analyses using simple asymptotic method were performed by calculating the relative excess risk due to interaction (RERI), attributable proportion because of interaction (AP) and synergy index (SI), and the additive interaction was considered statistically significant when the confidence interval of RERI, AP and SI did not include 0, 0, and 1, respectively.^32^

#### Sensitivity Analyses

To examine the robustness of the results, several sensitivity analyses were conducted (1) by changing knots in RCS analyses (Supplementary Fig. S3); (2) by adjusting surgical year in Cox models to mitigate statistical biases arising from advancements in surgical techniques, anti-fibrotic protocols, and postoperative care management (Supplementary Fig. S5); (3) by constructing random-effects Cox models fitting RCS curves for both unilateral and bilateral surgery patients, respectively (Supplementary Fig. S6); (4) by stratifying surgical failure by severity, including only those treated with postoperative antifibrotic drugs or only those requiring reoperation due to unsatisfactory outcomes, to demonstrate the robustness of the U-shaped association and the relatively low-risk interval (Supplementary Fig. S7); (5) by including those exposed to extreme ambient temperature (Supplementary Fig. S8); (6) by using the method of variance estimates recovery (MOVER) approach in the additive interaction analyses,^32^ instead of simple asymptotic method (Supplementary Table S10); and (7) by identifying ambient temperature as binary variable (Supplementary Tables S11 and S12) in interaction analyses.

#### Software and Statistical Significance

All analyses were conducted using R software (Version 4.0.2, R Foundation for Statistical Computing, Vienna, Austria). A two-tailed *P*-value < 0.05 was considered statistically significant.

## Results

### Participant Demographics and Clinical Characteristics

A total of 5193 participants were enrolled in this study (Fig. 1). Of these, surgical failure was observed in 215 (4.14%), 583 (11.2%), and 675 (13.0%) participants at 30, 90, and 180 days, respectively. Mean follow-up times for these intervals were 25.32, 57.62, and 93.38 days. Baseline characteristics, stratified by surgical outcome, are summarized in Supplementary Tables S2, S3, and Table 1. No significant differences were observed across ambient temperature tiers regarding age, sex, baseline IOP, preoperative medication duration, surgical type, geographic location, or glaucoma subtype. Those in higher temperature environments exhibited higher prevalence rates of hypertension and diabetes. Notably, however, ambient temperature variations did not significantly affect glaucoma surgical outcomes at any follow-up interval (30-day, *P* = 0.5793; 90-day, *P* = 0.4849; 180-day, *P* = 0.4609).

**Table 1.** Characteristics of Participants Exposed in Lower/Higher Average Ambient Temperature 180 Days Post-Surgery

Characteristic	Total (*N* = 5193)	Q1 (*N* = 2596)	Q2 (*N* = 2597)	*P* Value
Suboptimal outcomes				0.4609
Yes	675 (13.0)	347 (13.4)	328 (12.6)	
No	4518 (87.0)	2249 (86.6)	2269 (87.4)	
Type of failure				**0.0033** ^a^
IOP failure	622 (92.1)	309 (89.0)	313 (95.4)	
Reoperation/Revision	53 (7.9)	38 (11.0)	15 (4.6)	
Ambient temperature (°C)				**<0.0001** ^a^
Mean ± SD	16.50 ± 7.50	10.19 ± 4.79	22.80 ± 3.18	
Median (IQR)	16.94 (10.81–22.92)	10.81 (7.53–13.63)	22.92 (20.33–24.77)	
Range	−23.90∼33.29	−23.90∼16.93	16.94∼33.29	
Age (y)				0.7182
Mean ± SD	56.84 ± 14.35	56.95 ± 14.32	56.73 ± 14.39	
Median (IQR)	59.00 (49.00–67.00)	59.00 (49.00–68.00)	59.00 (48.00–67.00)	
Range	18.00∼86.00	18.00∼86.00	18.00∼86.00	
Baseline IOP (mm Hg)				0.1842
Mean ± SD	33.01 ± 11.52	33.16 ± 11.30	32.86 ± 11.73	
Median (IQR)	32.00 (23.00–42.00)	32.40 (23.80–42.00)	32.00 (22.80–42.00)	
Range	15.10∼73.00	15.10∼73.00	15.10∼72.00	
Preoperative eye drop duration (m)				0.8782
Mean ± SD	10.11 ± 24.81	10.21 ± 26.17	10.00 ± 23.38	
Median (IQR)	3.00 (1.00–11.00)	3.00 (1.00–12.00)	3.00 (1.00–10.00)	
Range	0.00∼600.00	0.00∼600.00	0.00∼360.00	
Sex				0.7466
Male	2906 (56.0)	1446 (55.7)	1460 (56.2)	
Female	2287 (44.0)	1150 (44.3)	1137 (43.8)	
Intraoperative agent				**<0.0001** ^a^
5-fluorouracil	1010 (19.4)	541 (20.8)	469 (18.1)	
Mitomycin C	2164 (41.7)	1123 (43.3)	1041 (40.1)	
No drug use	2019 (38.9)	932 (35.9)	1087 (41.9)	
Surgery type				0.1970
Trabeculectomy	2583 (49.7)	1305 (50.3)	1278 (49.2)	
Ex-PRESS shunt	731 (14.1)	343 (13.2)	388 (14.9)	
Ahmed glaucoma valve	1879 (36.2)	948 (36.5)	931 (35.8)	
Economic geographical location				0.9276
East	3956 (76.2)	1975 (76.1)	1981 (76.3)	
East-north	107 (2.1)	52 (2.0)	55 (2.1)	
Middle	993 (19.1)	497 (19.1)	496 (19.1)	
West	137 (2.6)	72 (2.8)	65 (2.5)	
Hypertension				**0.0016** ^a^
Yes	1373 (26.4)	636 (24.5)	737 (28.4)	
No	3820 (73.6)	1960 (75.5)	1860 (71.6)	
Diabetes				0.0647
Yes	846 (16.3)	398 (15.3)	448 (17.3)	
No	4347 (83.7)	2198 (84.7)	2149 (82.7)	
Glaucoma subtype				0.1545
POAG	1342 (25.8)	688 (26.5)	654 (25.2)	
PACG	2641 (50.9)	1285 (49.5)	1356 (52.2)	
Secondary glaucoma	1210 (23.3)	623 (24.0)	587 (22.6)	
Preoperative eye drop				
Prostaglandin analogs	1753 (33.8)	908 (35.0)	845 (32.5)	0.0704
Beta blockers	2178 (41.9)	1061 (40.9)	1117 (43.0)	0.1191
Alpha-2 agonists	2746 (52.9)	1393 (53.7)	1353 (52.1)	0.2605
Carbonic anhydrase inhibitors	2322 (44.7)	1124 (43.3)	1198 (46.1)	**0.0404** ^a^
Cholinergic agents	1259 (24.2)	616 (23.7)	643 (24.8)	0.3955
Fixed-combination drugs	171 (3.3)	84 (3.2)	87 (3.4)	0.8744
Anti-inflammatory drugs	571 (11.0)	271 (10.4)	300 (11.6)	0.2124
Unknown medications	388 (7.5)	190 (7.3)	198 (7.6)	0.7089
No medications	327 (6.3)	179 (6.9)	148 (5.7)	0.0872
Postoperative antifibrotic drugs				0.7745
Yes	903 (17.4)	456 (17.6)	447 (17.2)	
No	4290 (82.6)	2140 (82.4)	2150 (82.8)	
Surgery season				**<** **0.0001** ^a^
Spring	1587 (30.6)	396 (15.3)	1191 (45.9)	
Summer	1196 (23.0)	204 (7.9)	992 (38.2)	
Autumn	1050 (20.2)	841 (32.4)	209 (8.0)	
Winter	1360 (26.2)	1155 (44.5)	205 (7.9)	
Surgery year				**0.0121** ^a^
2015	505 (9.7)	266 (10.2)	239 (9.2)	
2016	560 (10.8)	266 (10.2)	294 (11.3)	
2017	526 (10.1)	257 (9.9)	269 (10.4)	
2018	509 (9.8)	254 (9.8)	255 (9.8)	
2019	602 (11.6)	333 (12.8)	269 (10.4)	
2020	527 (10.1)	248 (9.6)	279 (10.7)	
2021	547 (10.5)	254 (9.8)	293 (11.3)	
2022	426 (8.2)	204 (7.9)	222 (8.5)	
2023	657 (12.7)	326 (12.6)	331 (12.7)	
2024	334 (6.4)	188 (7.2)	146 (5.6)	

IQR, interquartile range; SD, standard deviation.

a *P* < 0.05.

### Potential Nonlinear Association Between Ambient Temperature and Surgical Failure

**Figure 2.**
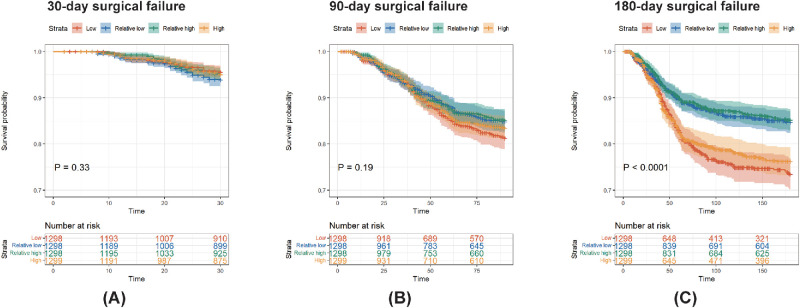
Kaplan-Meier survival analysis curves. They are for (**A**) 30-day, (**B**) 90-day, and (**C**) 180-day surgical failure, stratified by ambient temperature levels.

To assess the relationship between ambient temperature and surgical prognosis, participants were stratified into six groups based on postoperative temperature exposure. Histograms revealed a U-shaped association, which was most pronounced at the 180-day follow-up (Supplementary Fig. S1). Kaplan–Meier survival analyses further supported this finding, showing significantly higher risks of surgical failure at both temperature extremes, particularly at 180 days (*P* < 0.0001; Fig. 2). Multivariable random-effects Cox models were used to examine the potential independent association of ambient temperature. However, no statistically significant associations were observed between temperature and surgical failure at 30, 90, or 180 days, whether temperature was treated as a continuous or categorical variable (*P* > 0.05 or *P*_trend_ > 0.05 for all comparisons; Supplementary Table S4). These null findings warranted further exploration of potential nonlinear or threshold effects.

### Spline-Based Evaluation of Nonlinear Associations

**Figure 3.**
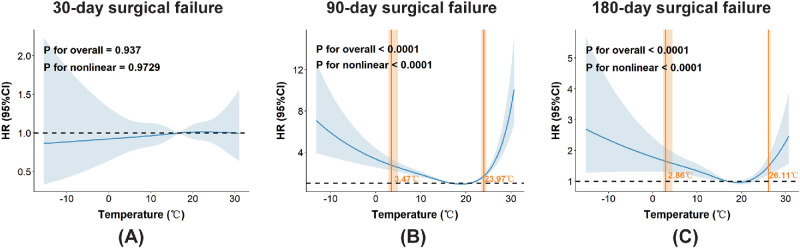
RCS curves of ambient temperature for (**A**) 30-day, (**B**) 90-day, and (**C**) 180-day surgical failure. RCS regression model was adjusted for age, sex, economic geographical location, comorbidities, glaucoma subtype, surgery season, preoperative ophthalmic solutions use, IOP, intraoperative agent and surgery type.

To address the observed U-shape, we conducted stratified Cox regressions by season, which showed that both winter cold and summer heat were consistently associated with elevated surgical failure risk (Supplementary Fig. S2). Consistent with the typical non-linear relationship often observed between environmental exposures and health outcomes, we used RCS to evaluate potential non-monotonic associations between intraoperative temperature and surgical failure risk. This is an exploratory and hypothesis-generating approach, employed precisely when a linear association is found to be nonsignificant. The RCS analysis revealed a significant U-shaped association at both 90- and 180-day follow-ups (non-linear *P* < 0.0001; Fig. 3). Model comparison demonstrated that the RCS model provided a superior fit to the data compared to the linear Cox specification (Supplementary Table S5), as evidenced by a highly significant likelihood ratio test (*P* < 0.001) and lower Akaike and Bayesian information criteria (ΔAIC and ΔBIC both < 0). Internal validation via bootstrapping yielded minimal optimism (optimism < 0.01), indicating negligible overfitting. Sensitivity analyses using alternative knot placements produced consistent curve shapes, reinforcing the robustness of the observed pattern (Supplementary Fig. S3). Collectively, these findings suggest that the nonsignificant linear association in the primary analysis likely reflects the cancellation of opposing risk effects at high and low temperatures within a single linear term. We therefore fitted random-effects Cox models on either side of the statistically identified point of minimum risk within the RCS model (Supplementary Table S6). The results demonstrated that increasing temperatures on the left side served as a protective factor, whereas those on the right side acted as a risk factor—consistent with the U-shaped association observed in the RCS curve.

Subsequently, we identified estimated modeled turning points within the spline curve on either side of the temperature associated with the lowest hazard. Specifically, for 90-day surgical failure, the model-derived left and right turning points were approximately 2.86°C and 26.11°C, respectively; for 180-day surgical failure, the corresponding points were 3.47°C and 23.97°C (Supplementary Table S7). To further validate the relevance of these ranges, we conducted a three-segment piecewise Cox regression analysis with random effects (Table 2). Different from our initial expectation, the U-shaped association between temperature and surgical failure was not symmetric. Below the left inflection point, the risk of surgical failure remained largely constant with further temperature decrease, rather than continuing to rise as observed on the high-temperature side. This pattern suggests that the statistical relationship between temperature extremes and surgical failure may be non-symmetric within the observed range.

**Table 2.** Three Segmented Random-Effects Cox Regression on 90- and 180-Day Surgical Failure

Temperature (°C)	HR (95% CI)	*P* Value
90-day suboptimal outcomes		
Temperature < 2.86°C	1.095 (0.967–1.239)	0.153
26.11°C > Temperature ≥ 2.86°C	0.938 (0.920–0.956)	**<0.0001** ^a^
Temperature ≥ 26.11°C	1.614 (1.338–1.946)	**<0.0001** ^a^
180-day suboptimal outcomes		
Temperature < 3.47°C	1.019 (0.912–1.139)	0.738
23.97°C > Temperature ≥ 3.47°C	0.910 (0.891–0.929)	**<0.0001** ^a^
Temperature ≥ 23.97°C	1.207 (1.079–1.349)	**0.0010** ^a^

The model was adjusted for age, sex, economic geographical location, comorbidities, glaucoma subtype, surgery season, preoperative ophthalmic solutions use, IOP, intraoperative agent and surgery type. The HRs indicates an increased risk for every degree Celsius rise in ambient temperature.

a *P* < 0.05.

### Stratification and Interaction Analyses

**Figure 4.**
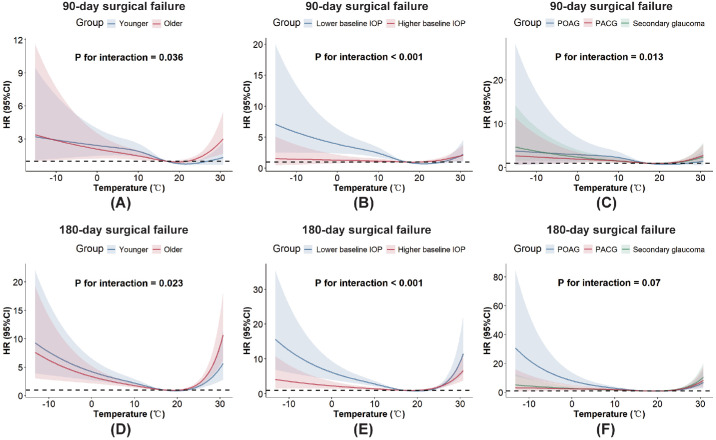
RCS curves of random-effects Cox models of various subgroups for ambient temperature at 90 (**A–C**) and 180 (**D–F**) days after surgery. Patients were respectively stratified by age (>60 years vs. 18–60 years, A/D), baseline IOP (high vs. low), and glaucoma subtype (PACG, POAG, secondary glaucoma, C/F).

Subgroup analyses at postoperative 90-day and 180-day follow-ups demonstrated consistent trends (Fig. 4). Older individuals (aged >60 years) and those diagnosed with POAG—rather than PACG or secondary glaucoma—were more susceptible to the adverse effects of extreme temperatures (either low or high). Lower baseline IOP was associated with an increased risk of surgical failure in cold environments. No significant effect modifications were observed for sex or intraoperative medication use (all *P* for interaction > 0.05; Supplementary Fig. S4).

### Sensitivity Analyses

Additional adjustment for surgery year did not alter the U-shaped association between ambient temperature and the risk of surgical failure, nor did it substantially affect the range of the low-risk interval (Supplementary Fig. S5). Results remained robust across patients undergoing unilateral or bilateral surgery (Supplementary Fig. S6). Stratification by outcome severity similarly preserved the U-shaped association between temperature and risk (Supplementary Fig. S7). Inclusion of previously excluded extreme temperature values did not alter the overall trend (Supplementary Fig. S8).

### Air Pollution as a Modifier of the Association Between Ambient Temperature and Surgical Outcomes

Given that previous studies have identified close relationship between air pollutants and ambient temperature,^10^^–^^15^^,^^33^^,^^34^ we further investigated the potential effect modification by air pollution on the nonlinear association between temperature and surgical outcomes. Supplementary Table S8 illustrated exposure levels of six air pollutants for different outcomes. Single-pollutant models demonstrated a significant association of PM_2.5_, PM_10_, NO_2_ and CO with surgical failure (Supplementary Table S9). To assess the independent role of ambient temperature, we adjusted for air pollutants in Cox proportional hazards regression models across different postoperative durations. Adjustment for air pollution significantly altered the HRs for ambient temperature (Table 3), indicating a potential interaction. No multicollinearity was observed, with VIF less than five (data not shown).

**Table 3.** Cox Proportional Hazard Ratios for Temperature

	Model 1		Model 2		Model 3		Model 4	
Temperature (°C) Continuous Variable Per 5°C	HR (95% CI)	*P* Value	HR (95% CI)	*P* Value	HR (95% CI)	*P* Value	HR (95% CI)	*P* Value
30-day suboptimal outcome							
PM_2.5_	1.144 (1.062–1.233)	**0.0004** ^a^	1.136 (1.054–1.224)	**0.0008** ^a^	1.137 (1.055–1.225)	**0.0008** ^a^	1.123 (1.040–1.213)	**0.0032** ^a^
PM_10_	1.095 (1.025–1.170)	**0.0074** ^a^	1.082 (1.013–1.156)	**0.0188** ^a^	1.083 (1.014–1.157)	**0.0177** ^a^	1.066 (0.997–1.139)	0.0629
NO_2_	1.094 (1.025–1.168)	**0.0067** ^a^	1.101 (1.032–1.176)	**0.0038** ^a^	1.102 (1.032–1.176)	**0.0037** ^a^	1.098 (1.027–1.173)	**0.0062** ^a^
SO_2_	1.024 (0.969–1.082)	0.3936	1.020 (0.965–1.078)	0.4808	1.021 (0.966–1.078)	0.4712	1.009 (0.954–1.067)	0.7515
O_3_	0.981 (0.909–1.059)	0.6297	0.976 (0.904–1.053)	0.5275	0.976 (0.904–1.053)	0.5295	0.972 (0.901–1.048)	0.4539
CO	1.061 (1.000–1.125)	**0.0498** ^a^	1.057 (0.997–1.122)	0.0638	1.058 (0.997–1.122)	0.0621	1.050 (0.989–1.114)	0.1125
90-day suboptimal outcome							
PM_2.5_	1.077 (1.004–1.156)	**0.0379** ^a^	1.080 (1.006–1.159)	**0.0325** ^a^	1.080 (1.006–1.159)	**0.0327** ^a^	1.073 (0.997–1.154)	0.0607
PM_10_	1.038 (0.975–1.106)	0.2426	1.036 (0.972–1.103)	0.278	1.035 (0.972–1.103)	0.2788	1.022 (0.959–1.090)	0.4949
NO_2_	1.029 (0.968–1.094)	0.3573	1.039 (0.977–1.106)	0.2189	1.039 (0.977–1.105)	0.2199	1.042 (0.978–1.110)	0.2058
SO_2_	0.984 (0.932–1.038)	0.5558	0.985 (0.934–1.040)	0.5906	0.985 (0.934–1.040)	0.5893	0.980 (0.928–1.035)	0.4622
O_3_	0.927 (0.860–0.999)	**0.0462** ^a^	0.925 (0.858–0.997)	**0.0405** ^a^	0.925 (0.858–0.997)	**0.0403** ^a^	0.922 (0.856–0.993)	**0.0316** ^a^
CO	1.007 (0.951–1.066)	0.8128	1.008 (0.953–1.067)	0.7749	1.008 (0.953–1.067)	0.7765	1.005 (0.949–1.065)	0.8665
180-day suboptimal outcome							
PM_2.5_	1.086 (1.009–1.170)	**0.028** ^a^	1.098 (1.019–1.184)	**0.0138** ^a^	1.099 (1.019–1.184)	**0.0137** ^a^	1.094 (1.013–1.182)	**0.0223** ^a^
PM_10_	1.047 (0.979–1.121)	0.1776	1.051 (0.983–1.125)	0.1461	1.051 (0.983–1.125)	0.1462	1.038 (0.969–1.111)	0.2878
NO_2_	1.011 (0.948–1.078)	0.7415	1.018 (0.954–1.086)	0.5848	1.018 (0.954–1.086)	0.586	1.024 (0.958–1.094)	0.4863
SO_2_	0.978 (0.923–1.037)	0.4588	0.980 (0.925–1.039)	0.5039	0.980 (0.925–1.039)	0.5024	0.976 (0.920–1.035)	0.4139
O_3_	0.903 (0.834–0.978)	**0.012** ^a^	0.897 (0.829–0.972)	**0.0078** ^a^	0.897 (0.828–0.972)	**0.0076** ^a^	0.895 (0.826–0.969)	**0.006** ^a^
CO	1.004 (0.944–1.067)	0.9088	1.006 (0.946–1.069)	0.8517	1.006 (0.946–1.069)	0.8533	1.004 (0.944–1.068)	0.9036

PM_2.5_, particulate matter with an aerodynamic diameter less than 2.5 µm; PM_10_, particulate matter with an aerodynamic diameter less than 10 µm; Q, quartile.

Model 1: Adjusted for specific pollutant; Model 2: Model 1 additionally adjusted for age, sex, economic geographical location, comorbidities and glaucoma subtype; Model 3: Model 2 additionally adjusted for preoperative ophthalmic solutions use; Model 4: Model 3 additionally adjusted for surgery season, IOP, intraoperative agent, and surgery type.

a *P* < 0.05.

When PM_2.5_ exposure was adjusted, the risks of ambient temperature for 30-, 90-, and 180-day surgical failure were significant (*P* < 0.05 for all). Additionally, the adjustment of O_3_ was associated with changes in the observed relationship with outcomes 90 and 180 days after surgery, and the inclusion of PM_10_ or NO_2_ resulted in a statistically significant association between ambient temperature and surgical failure at 30 days after surgery. Supplementary Figure S9 described the correlation between temperature and air pollutants. RCS curves for the single-pollutant models demonstrated the similar trend of ambient temperature with previous models, but the additional adjustment for PM_2.5_, PM_10_ and CO specifically reduced the risk of ambient temperature at lower temperatures while elevating the risks at higher temperatures (Supplementary Fig. S10).

**Figure 5.**
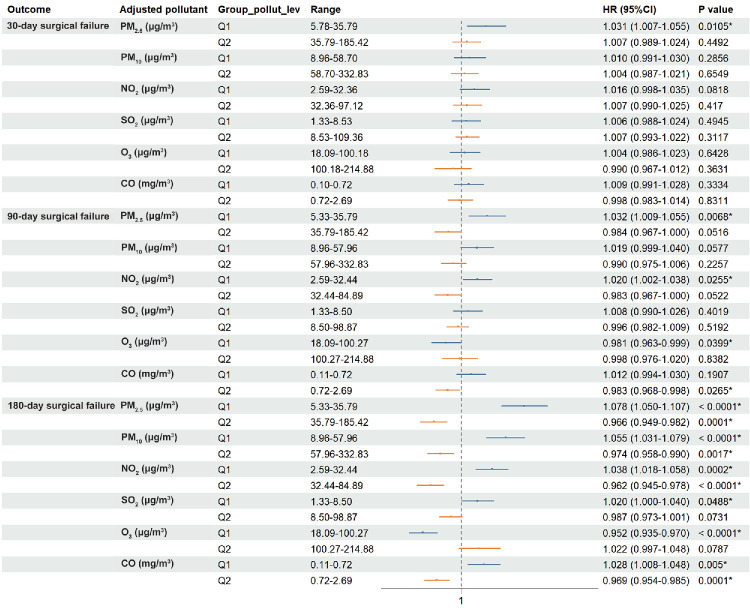
A forest plot depicting Cox proportional hazard ratios for temperature within various exposure levels of air pollutants. The models were adjusted for age, sex, economic geographical location, comorbidities, glaucoma subtype, surgery season, preoperative ophthalmic solutions use, IOP, intraoperative agent and surgery type. PM_2.5_, particulate matter with an aerodynamic diameter less than 2.5 µm; PM_10_, particulate matter with an aerodynamic diameter less than 10 µm; Q, quartile.

To further explore how pollutant concentrations modify the temperature-associated risks, we stratified participants based on median air pollution exposure levels. In low-pollution environments, rising ambient temperatures were positively correlated with the risk of surgical failure (Fig. 5). The divergence in temperature-related risks between high- and low-pollution groups widened over time, suggesting a cumulative interaction on long-term surgical prognosis.

### Interactive Effect of Ambient Temperature and Air Pollution

Based on preliminary findings that temperature impacts postoperative outcomes differently across air pollution levels, we conducted interaction analyses to further investigate the statistical interactions between these variables. Table 4 illustrates significant multiplicative interactions (*P*_interaction_ < 0.05) between ambient temperature strata and all pollutants within 180 days after surgery. With the exception of O_3_, all pollutants exhibited negative multiplicative interactions with ambient temperature. Additive interaction analyses mirrored this pattern, yielding RERI < 0, AP < 0, and SI < 1. The observed negative RERI indicates that the magnitude of the association between cold temperatures and higher risk was greater in the presence of higher pollution levels. These conclusions were robust across sensitivity analyses, including the “MOVER” method and the categorization of ambient temperature as a binary variable (Supplementary Tables S10–S12).

**Table 4.** Interaction Analyses With the Additive Interaction Analyses Using Simple Asymptotic Method

Outcomes and Adjusted Pollutant	Multiplicative Scale	P For Multiplicative Interaction	RERI (95% CI)	AP (95% CI)	SI (95% CI)
30-day suboptimal outcome				
PM_2.5_ (µg/m^3^)	0.98 (0.95–1.01)	0.7537	−0.01 (−0.05∼0.02)	−0.01 (−0.02∼0.01)	0.99 (0.96–1.02)
PM_10_ (µg/m^3^)	0.99 (0.97–1.02)	0.6459	−0.00 (−0.03∼0.02)	−0.00 (−0.02∼0.02)	0.99 (0.93–1.05)
NO_2_ (µg/m^3^)	1.00 (0.97–1.02)	0.7960	0.00 (−0.02∼0.02)	0.00 (−0.02∼0.02)	1.00 (0.93–1.07)
SO_2_ (µg/m^3^)	1.00 (0.98–1.02)	0.9202	0.00 (−0.02∼0.02)	0.00 (−0.01∼0.02)	1.01 (0.92–1.11)
O_3_ (µg/m^3^)	0.99 (0.96–1.02)	0.3800	−0.02 (−0.06∼0.03)	−0.01 (−0.04∼0.01)	0.96 (0.92–1.00)
CO (mg/m^3^)	0.99 (0.97–1.01)	0.3459	−0.01 (−0.04∼0.01)	−0.01 (−0.03∼0.01)	0.95 (0.89–1.01)
90-day suboptimal outcome				
PM_2.5_ (µg/m^3^)	0.96 (0.93–0.98)	**0.0013** ^a^	**−0.07 (**−**0.13∼**−**0.00)**^b^	−**0.03 (**−**0.04∼**−**0.01)**^c^	**0.96 (0.94**–**0.98)**^d^
PM_10_ (µg/m^3^)	0.97 (0.94–0.99)	**0.0105** **^a^**	−**0.05 (**−**0.10∼0.00)**^b^	−**0.02 (**−**0.04∼**−**0.01)**^c^	**0.96 (0.94**–**0.98)**^d^
NO_2_ (µg/m^3^)	0.97 (0.94–0.99)	**0.0060** **^a^**	−**0.05 (**−**0.10∼**−**0.00)** ^b^	−**0.03 (**−**0.04∼**−**0.01)**^c^	**0.95 (0.93**–**0.97)**^d^
SO_2_ (µg/m^3^)	0.98 (0.96–1.01)	0.1566	−0.03 (**−**0.06∼0.01)	−**0.01 (**−**0.03∼0.00)**^c^	**0.97 (0.95**–**1.00)**^d^
O_3_ (µg/m^3^)	1.02 (0.99–1.05)	0.1263	0.02 (−0.00∼0.05)	0.03 (−0.02∼0.07)	NA
CO (mg/m^3^)	0.97 (0.95–0.99)	**0.0124** **^a^**	−**0.04 (**−**0.08∼0.00)**^b^	−**0.03 (**−**0.04∼**−**0.01)**^c^	**0.94 (0.91**–**0.97)**^d^
180-day suboptimal outcome				
PM_2.5_ (µg/m^3^)	0.90 (0.87–0.93)	**<0.0001** **^a^**	−**0.35 (**−**0.62∼**−**0.08)**^b^	−**0.04 (**−**0.06∼**−**0.03)**^c^	**0.95 (0.94**–**0.97)**^d^
PM_10_ (µg/m^3^)	0.92 (0.90–0.95)	**<0.0001** **^a^**	−**0.20 (**−**0.35∼**−**0.05)**^b^	−**0.04 (**−**0.05∼**−**0.02)**^c^	**0.96 (0.94**–**0.97)**^d^
NO_2_ (µg/m^3^)	0.93 (0.90–0.95)	**<0.0001** **^a^**	−**0.19 (**−**0.31∼**−**0.06)**^b^	−**0.05 (**−**0.07∼**−**0.03)**^c^	**0.94 (0.92**–**0.95)**^d^
SO_2_ (µg/m^3^)	0.96 (0.94–0.99)	**0.0017** **^a^**	−**0.07 (**−**0.13∼**−**0.01)**^b^	−**0.03 (**−**0.04∼**−**0.01)**^c^	**0.96 (0.94**–**0.98)**^d^
O_3_ (µg/m^3^)	1.08 (1.05–1.11)	**<0.0001** **^a^**	0.06 (0.04–0.07)^b^	0.15 (0.04–0.27)^c^	NA
CO (mg/m^3^)	0.94 (0.92–0.96)	**<0.0001** **^a^**	−**0.12 (**−**0.21∼**−**0.04)**^b^	−**0.04 (**−**0.06∼**−**0.03)**^c^	**0.93 (0.92**–**0.95)**^d^

PM_2.5_, particulate matter with an aerodynamic diameter less than 2.5 µm; PM_10_, particulate matter with an aerodynamic diameter less than 10 µm.

The model was adjusted for specific air pollutant, age, sex, economic geographical location, comorbidities, glaucoma subtype, surgery season, preoperative ophthalmic solutions use, IOP, intraoperative agent and surgery type. In the model, air pollutant was considered as a binary variable. Both multiplicative and additive interaction analyses were performed. Simple asymptotic method was selected to conduct additive interaction analyses.

a *P* < 0.05.

b RERI < 0.

c AP < 0.

d SI < 1.

## Discussion

This large, retrospective cohort study is the first to investigate the potential association between ambient temperature and postoperative outcomes following incisional glaucoma surgery. Through nonlinear modeling, we identified a distinct U-shaped, asymmetric relationship, in which both lower and higher ambient temperatures were associated with an increased risk of surgical failure under these specific analytical specifications. A negative interaction between ambient temperature and pollutant exposure was observed, suggesting a complex joint association of climate and environmental factors with glaucoma surgical outcomes. These findings point to a possible link between environmental factors, particularly ambient temperature, and postoperative prognosis within a nonlinear framework.

### Climate Change and Narrowing Thermal Safety Zones

Climate change has intensified the frequency, severity, and duration of extreme weather events, including both heatwaves^35^ and cold spells. Although global temperatures are rising, cold extremes remain prevalent. This is partly driven by large-scale patterns like the negative North Atlantic Oscillation, which induces colder, drier winters across Northern^36^ and Southern^37^ Europe. The incidence of cold spells has also risen since 1979.^38^ Against the backdrop of increasing global frequency of extreme temperature events, climate information assumes its own significance in perioperative planning for glaucoma filtration surgery. Our findings indicate that temperatures below approximately 3°C maintain the risk of surgical failure at a consistently high level, whereas temperatures above the range of approximately 24°C–26°C lead to a significant increase in failure risk. With global climate change accelerating, this zone of climatic safety is narrowing. The number of days falling outside this range is increasing in many regions. However, it is crucial to clarify that all such thresholds are modeling constructs intended to facilitate clinical interpretation, and should not be interpreted as definitive biological boundaries.

### Delayed and Cumulative Association Between Thermal Exposure and Surgical Failure

Although most research has addressed the acute health impacts of extreme temperature events—such as heatwaves or cold spells—these studies typically focus on systemic cardiovascular or respiratory outcomes. However, evidence regarding temperature-related surgical risks in chronic ophthalmic diseases remains scarce, particularly concerning the full spectrum of ambient temperature exposure. Our study bridges this gap by characterizing the nonlinear, time-varying relationship between temperature and surgical outcomes. We identified delayed, cumulative effects, with the most pronounced associations emerging at 90 and 180 days after surgery. These findings are consistent with previous findings showing that temperature-related physiologic effects on health tend to emerge over longer exposure windows and follow a nonlinear trajectory.^39^

### Biological Mechanisms Underlying Thermal Vulnerability

Several biological pathways may explain the observed associations. In the context of glaucoma surgery, the direct evidence linking these pathways to conjunctival wound healing remains limited; however, plausible mechanisms can be inferred from broader ophthalmological and physiological studies. Cold exposure is known to reduce intraocular temperature,^40^ which can increase IOP^41^ by impairing aqueous humor dynamics. Seasonal studies from China have reported higher IOP in patients with POAG during winter months,^42^ likely because of reduced aqueous flow viscosity at lower temperatures.^4^^,^^5^ In addition, cold-induced vasoconstriction may compromise ocular perfusion and oxygen delivery, contributing to local hypoxia.^43^ When occurring during the early postoperative period, these physiological changes may impair conjunctival wound healing and trigger fibrotic responses at the surgical site.

In contrast, experimental data from non-ocular systems have shown that heat stress upregulates cytokines such as transforming growth factor-beta (TGF-β), interleukins (e.g., IL-6),^6^ and heat shock proteins,^7^ all of which are implicated in scar formation. TGF-β, in particular, promotes fibroblast-to-myofibroblast differentiation, driving extracellular matrix deposition and tissue remodeling.^44^ Higher temperatures may also enhance macrophage infiltration at the wound site, further amplifying local cytokine production and fibroblast activation.^45^ Although direct evidence in conjunctival fibroblasts is sparse, previous studies suggest that fibroblast proliferation and collagen synthesis are temperature-sensitive and can be enhanced in warmer environments.^7^ It is therefore plausible that elevated temperatures may similarly exacerbate conjunctival fibrosis by amplifying proinflammatory signaling, macrophage infiltration, and fibroblast activity at the bleb site.

### Age, POAG, and Thermal Susceptibility

Our findings support an environmentally responsive risk stratification model, identifying older adults and POAG patients as particularly vulnerable to temperature extremes. Although some studies have suggested increased postoperative fibrosis in younger individuals,^46^ ageing is associated with impaired thermoregulation due to decreased receptor sensitivity and prolonged temperature equilibration.^47^ This systemic vulnerability may extend to the ocular surface, potentially reducing the capacity to buffer against thermal stress. This may lead to reduced capacity to buffer against thermal stress, predisposing older individuals to hypoperfusion and localized hypoxia during cold exposure. Additionally, age-related declines in antioxidant defense mechanisms^48^ may potentiate oxidative stress, activating the NF-κB pathway and promoting TGF-β1–mediated fibrotic remodeling.^49^

POAG patient group often exhibits chronic vascular dysfunction, including elevated plasma endothelin, reduced nitric oxide bioavailability, and impaired vasodilation.^50^ We speculate that this compromised vascular baseline heightens their susceptibility to thermal insults. Under cold, excessive vasoconstriction could critically reduce ocular blood flow. Under heat, an inadequate vasodilatory reserve may fail to meet increased metabolic demand. In both scenarios, a potential consequence is tissue hypoxia. Hypoxia is a potent inducer of fibrosis in various organs, primarily through NF-κB activation. Thus hypoxia may represent a final common pathway linking thermal extremes to conjunctival scarring in this vulnerable population, though this specific sequence in the filtering bleb warrants direct experimental confirmation.

### Polluted Air Amplifies Cold-Related Risk in Glaucoma Surgery

Our analysis revealed a significant antagonistic interaction between elevated levels of particulate matter (PM_2.5_, PM_10_), NO_2_, SO_2_, CO and increased ambient temperature, consistent with a growing body of evidence.^33^^,^^34^ These results suggest that the magnitude of the association between low temperatures and surgical failure is significantly greater in the presence of higher air pollution. Conversely, under conditions of high pollution, the association between high temperatures and surgical failure becomes less pronounced—a finding that appears clinically counterintuitive.

The observed heterogeneity in interaction patterns may be attributed to distinct seasonal atmospheric dynamics. In winter, low temperatures frequently coincide with temperature inversions. These inversion layers trap pollutants, leading to elevated ground-level concentrations.^51^ In this setting, cold temperatures and high pollutant concentrations frequently co-occur, which is reflected statistically as an additive interaction regarding surgical outcomes. Conversely, during hot and heavily polluted summer conditions, the scattering and absorption of solar radiation by high aerosol concentrations (e.g., PM_2.5_) may reduce the net radiative flux reaching the surface.^52^ Consequently, even when ambient temperature readings are high, the actual radiant heat load experienced by patients could differ from measured ambient temperatures due to atmospheric aerosol shielding. Thus these interaction findings should be interpreted as statistical reflections of seasonal atmospheric stability and shifts in the combined influence of environmental stressors. However, the observed interaction may also reflect residual confounding by unmeasured meteorological factors or differential exposure misclassification, underscoring the need for future studies using a more comprehensive exposomics approach.

### Strengths and Limitations

This study has several strengths. It is, to our knowledge, the largest cohort to examine the relationship between ambient temperature and glaucoma surgical outcomes. The use of high-resolution satellite-derived environmental data, combined with advanced modeling techniques—including restricted cubic splines and segmented regression—allowed for nuanced characterization of nonlinear and delayed effects. Beside, cohorts from two institutions ensured generalizability.

However, limitations warrant consideration. The most significant limitation of this study is the lack of external validation in ethnically and geographically diverse cohorts. This precludes definitive conclusions about the universal applicability of our model/findings. The retrospective nature of the study may increase bias, although internal consistency was enhanced by uniform clinical management. In addition, data on bleb morphology (e.g., from anterior segment OCT) and medication adherence were unavailable, and socioeconomic data were limited to geographic proxies. Additionally, this mechanistically plausible U-shaped relationship between temperature and surgical outcome requires prospective validation. Finally, although satellite-derived exposures have been validated in large-scale health studies and remain the standard in environmental epidemiology,^16^^,^^53^ this approach does not account for individual-level behaviors such as indoor heating or air conditioning, time spent outdoors, workplace environment, or postoperative activity restrictions. Therefore outdoor temperature should be viewed as a standardized, population-level indicator of environmental thermal conditions rather than a direct measure of ocular surface temperature. Despite this limitation, outdoor temperature retains population-level relevance because of partial indoor-outdoor coupling and variable outdoor exposure from daily activities; and temperature plausibly modulates wound healing via biological pathways like inflammation and vascular responses. Future studies incorporating direct ocular surface monitoring or individual time-activity data are needed to refine exposure assessment and strengthen causal inference.

In this large cohort study, under nonlinear modeling specifications, non-optimal ambient temperatures were associated with an increased risk of surgical failure following incisional glaucoma surgery, exhibiting a U-shaped pattern. Given that conventional linear analyses were null, these findings suggest that the relationship between climate factors and ocular health may be complex and model-dependent. As global climate change and the frequency of temperature extremes continue to shift, further research is warranted to better understand the potential associations between environmental changes and postoperative outcomes.

## Supplementary Material

Supplement 1
