# Supplementary material for: Postoperative Risk and Climate Exposure: A Retrospective Study of Incisional Glaucoma Surgery Outcomes
Source: Transl Vis Sci Technol. 2026 Apr 27;15(4):24. doi: 10.1167/tvst.15.4.24 (PMC13112496; doi:10.1167/tvst.15.4.24)
Supplement: Supplement 1 [file tvst-15-4-24_s001.docx]

**Postoperative Risk and Climate Exposure: A Retrospective Cohort Study of Incisional Glaucoma Surgery Outcomes**

Tianhao Chen, Yi Tian, Yixiang Zhu, Jiaying Li, Haidong Kan, Xinghuai Sun^*^, Yuyan Zhang^*^, Yuan Lei^*^

^*^ Corresponding authors

**Contents**

Online supplemental figures (S1 to S10)........................................................2

Online supplemental tables (S1 to S12)..........................................................12


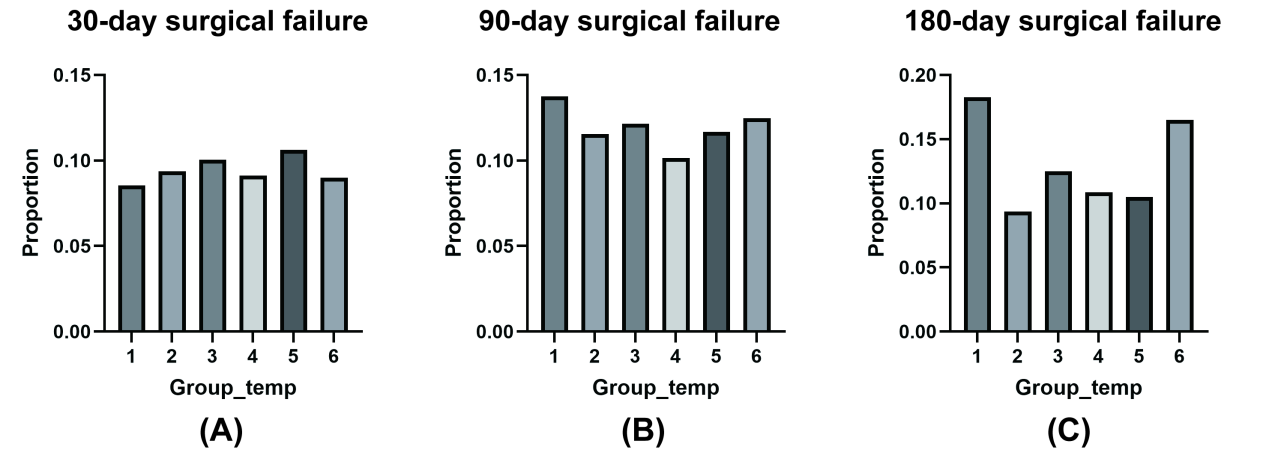


**Figure S1. The proportion of suboptimal surgical outcomes within (A) 30, (B) 90, and (C) 180 days post-surgery under different temperature levels**. Below the horizontal axis, 1-6 respectively represents for average ambient temperature level of -20 °C to 5 °C, 5 °C to 10 °C, 10 °C to 15 °C, 15 °C to 20 °C, 20 °C to 25 °C, 25 °C to 30 °C. The percentage of individuals with suboptimal surgical outcomes was calculated as the proportion of the total number of people within that group.

**
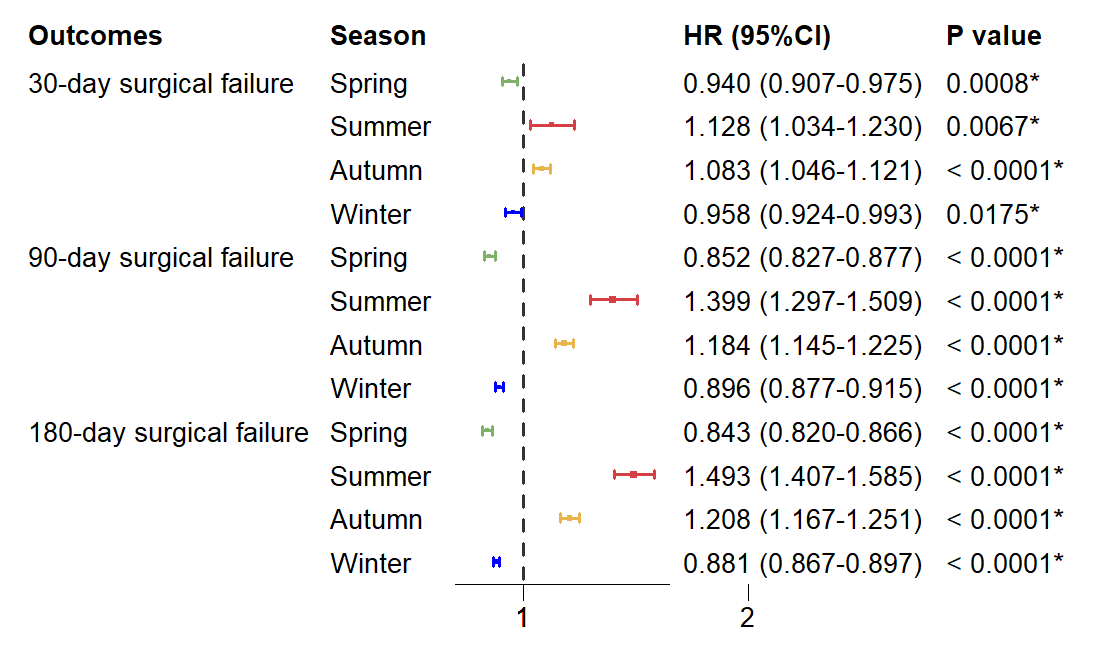
**

**Figure S2. A forest plot depicting random-effects Cox regression for temperature within various seasons**. The models were adjusted for age, sex, economic geographical location, comorbidities, glaucoma subtype, surgery season, preoperative ophthalmic solutions use, IOP, intraoperative agent and surgery type. IOP, intraocular pressure; HR, hazard ratio; CI, confidence interval.

**
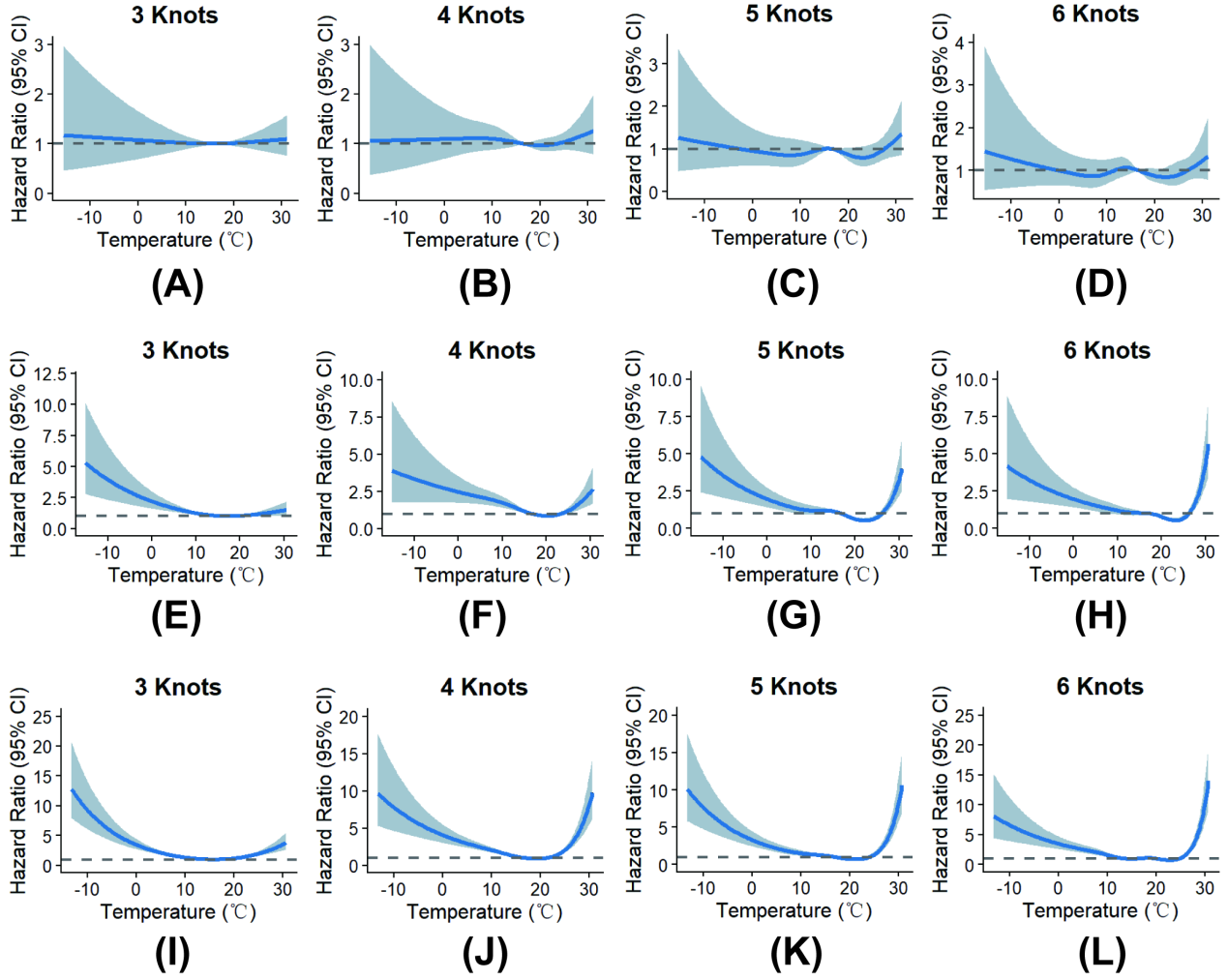
**

**Figure S3. RCS curves of sensitivity analyses changing fitting knots at 30 (A-D), 90 (E-H), and 180 (I-L) days post-surgery.** RCS, restricted cubic spline; HR, hazard ratio; CI, confidence interval.

**
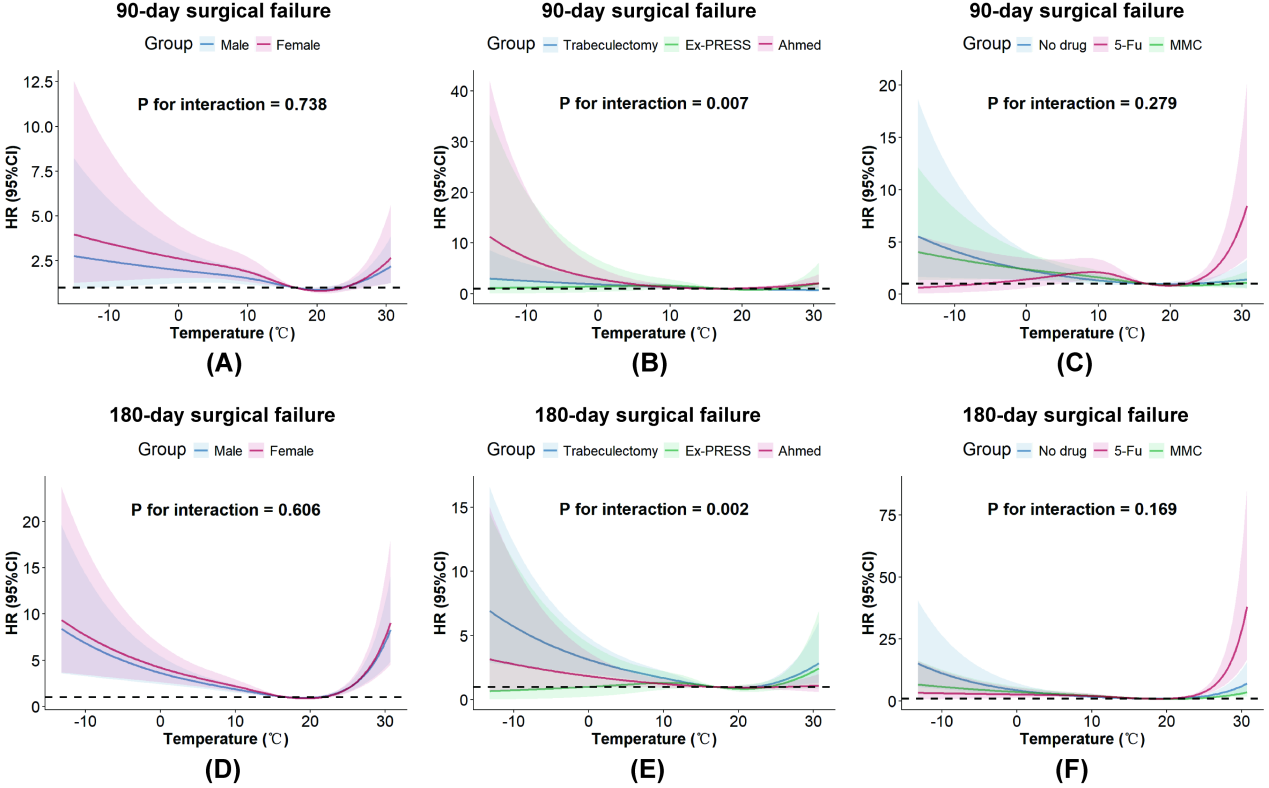
**

**Figure S4. RCS curves of random-effects Cox models of various subgroups for ambient temperature at 90 (A–C) and 180 (D–F) days post-surgery**. Patients were stratified by gender (female vs. male, A/D), surgery type (Ex-PRESS, trabeculectomy, Ahmed, B/E), and intraoperative agent (MMC, 5-Fu, no drug use, C/F). RCS, restricted cubic spline; MMC, mitomycin C; 5-Fu, 5-fluorouracil; HR, hazard ratio; CI, confidence interval.

**
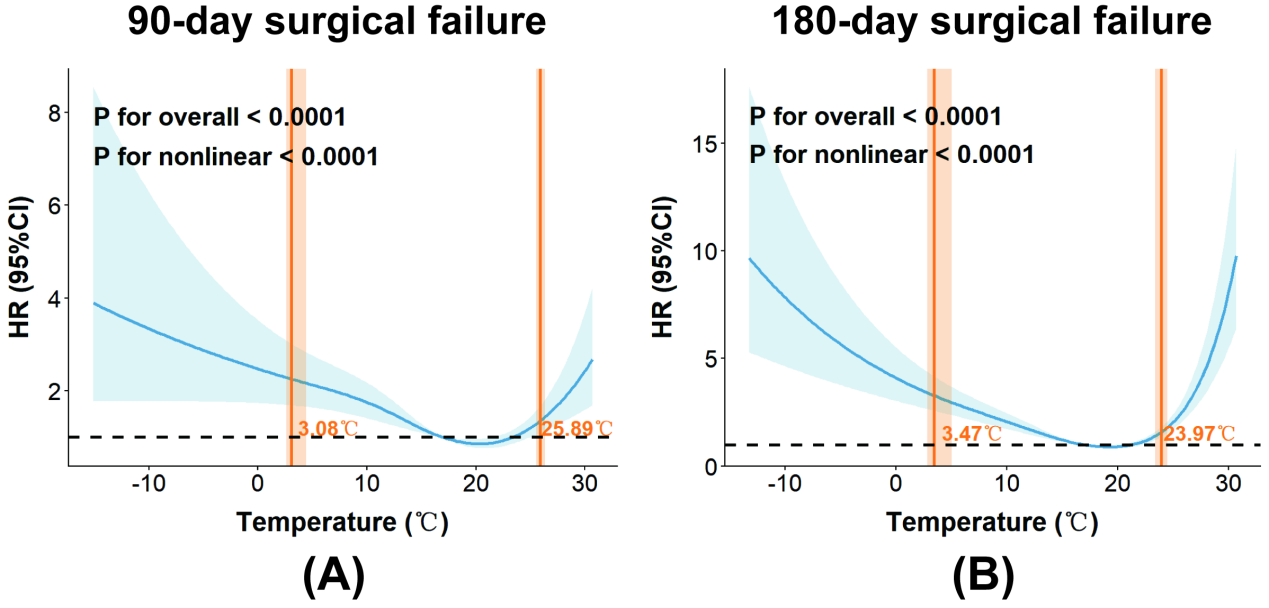
**

**Figure S5. RCS curves of random-effects Cox models additionally adjusted for surgery year to mitigate statistical biases**. RCS, restricted cubic spline; HR, hazard ratio; CI, confidence interval.

**
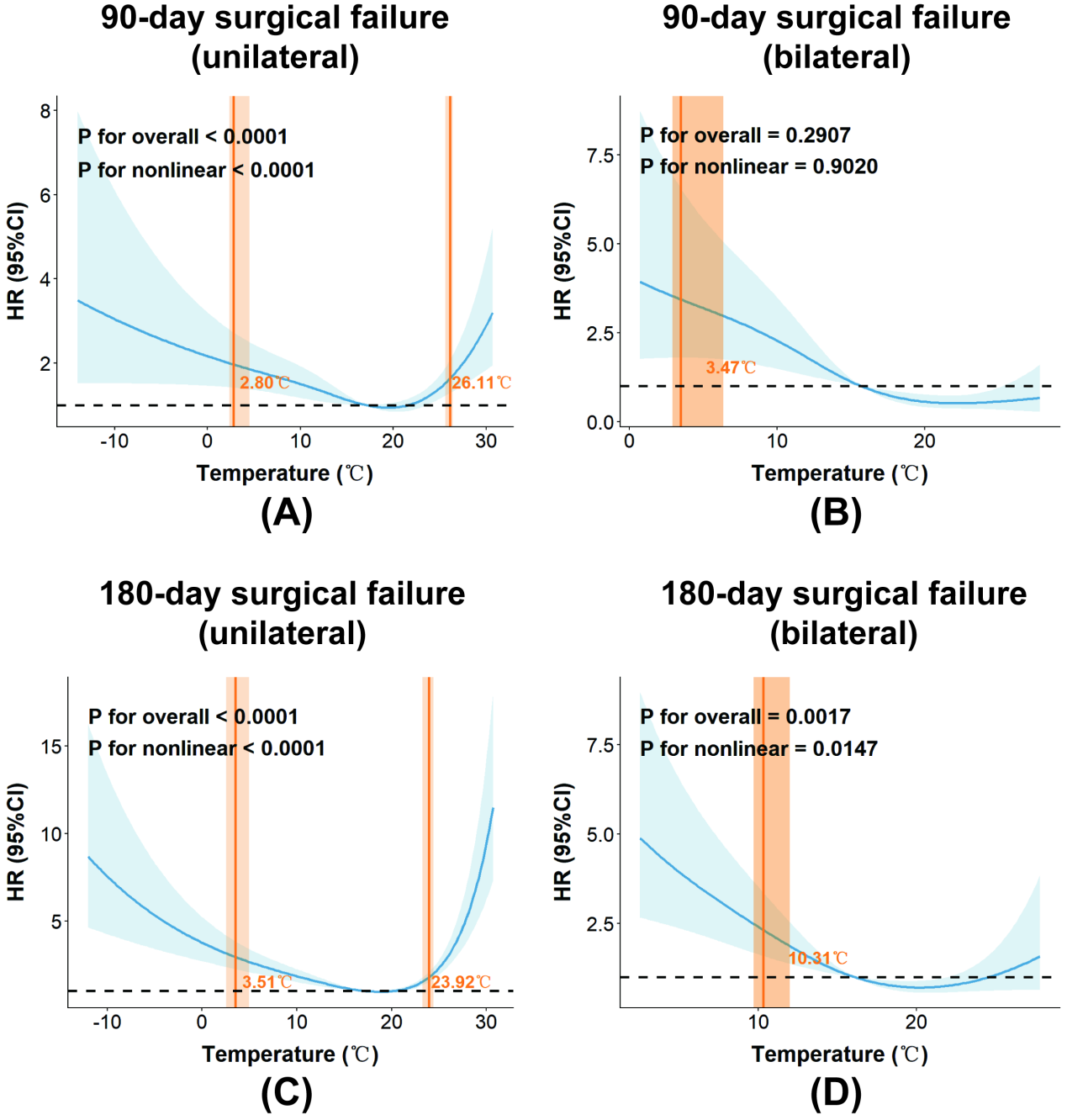
**

**Figure S6.** **RCS curves of Cox regression models for unilateral (A/B) and bilateral (C/D) cases at 90 (A/C) and 180 (B/D) days postoperatively.** RCS, restricted cubic spline; HR, hazard ratio; CI, confidence interval.

**
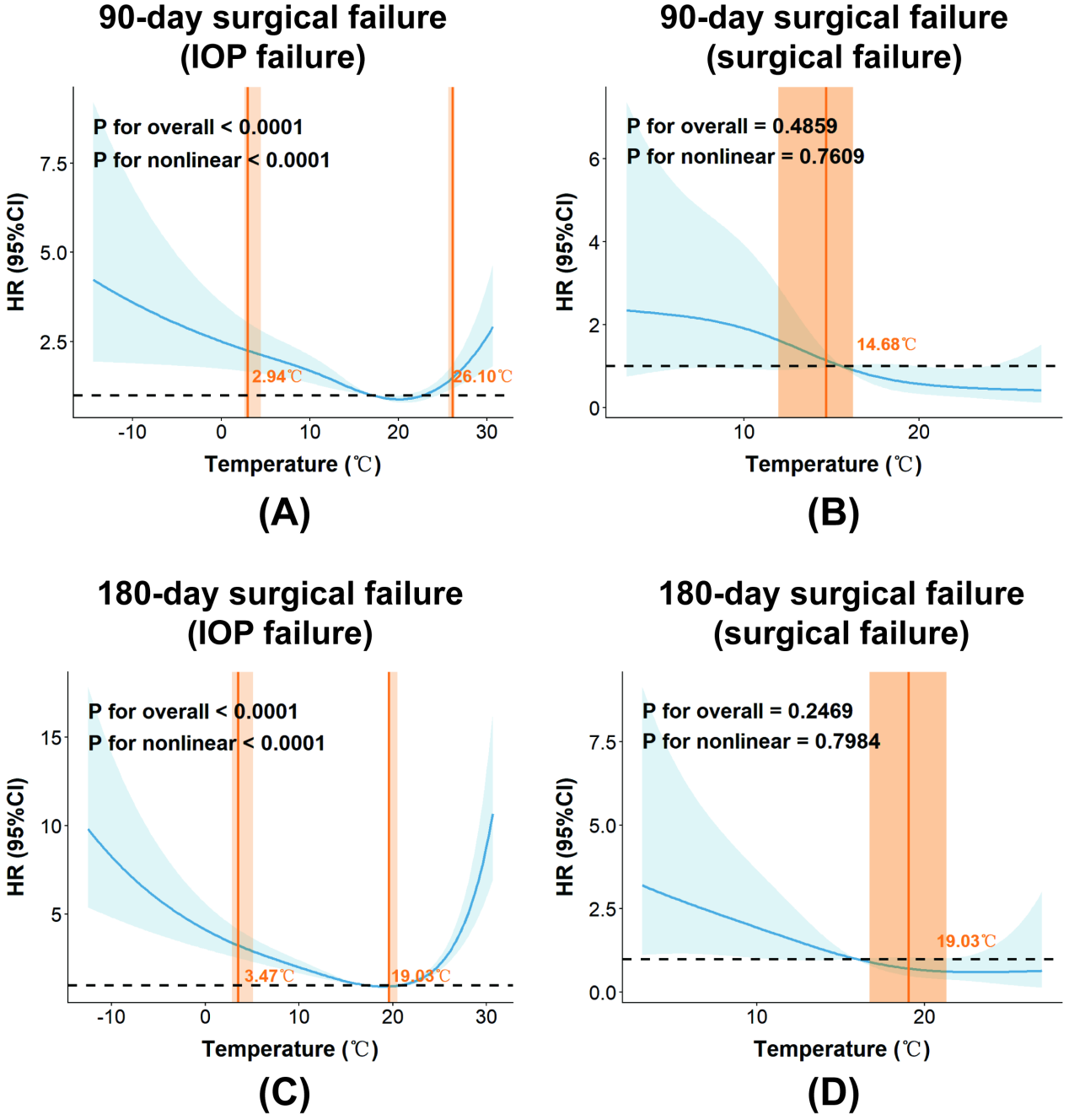
**

**Figure S7.** **RCS curves of Cox regression models for cases experiencing IOP failure (A/B) and reoperation cases (C/D) at 90 (A/C) and 180 (B/D) days postoperatively**. RCS, restricted cubic spline; HR, hazard ratio; CI, confidence interval.

**
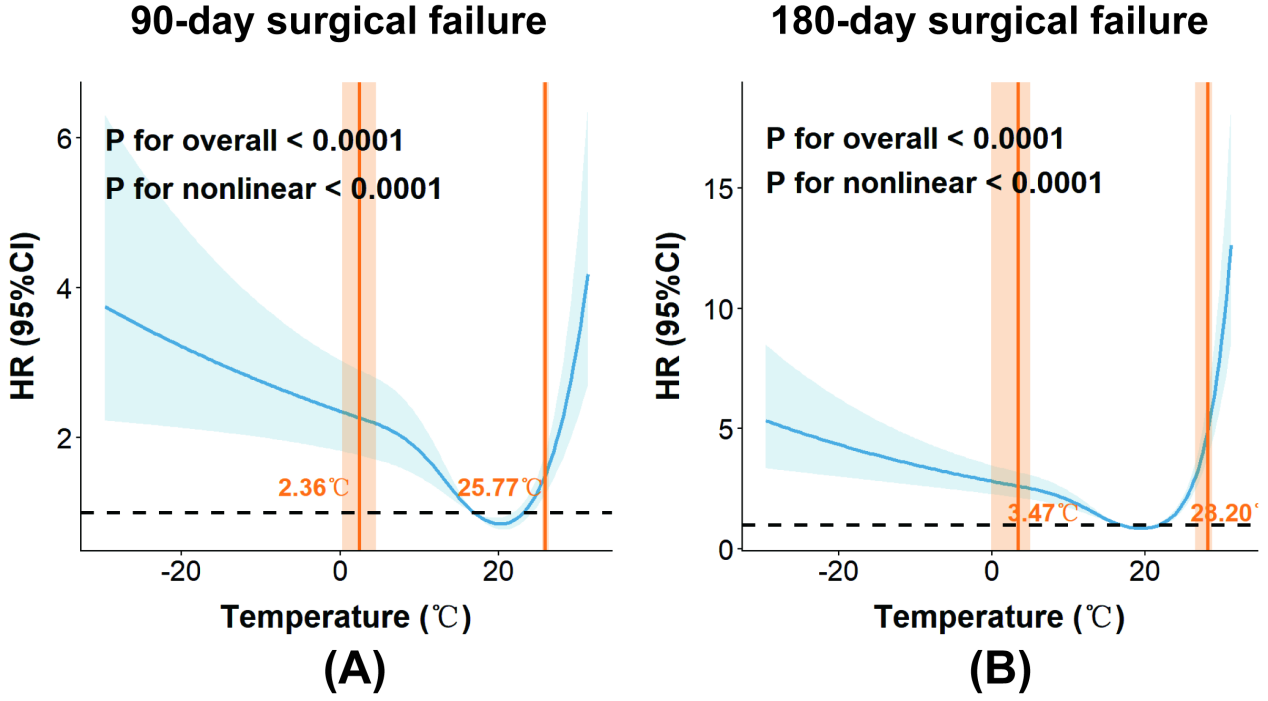
**

**Figure S8. RCS curves of Cox regression models for cohort not excluding those exposed to extreme cold (lower than the 2.5th location-specific percentile) or extreme hot temperature (higher than the 97.5th location-specific percentile).** RCS, restricted cubic spline; HR, hazard ratio; CI, confidence interval.


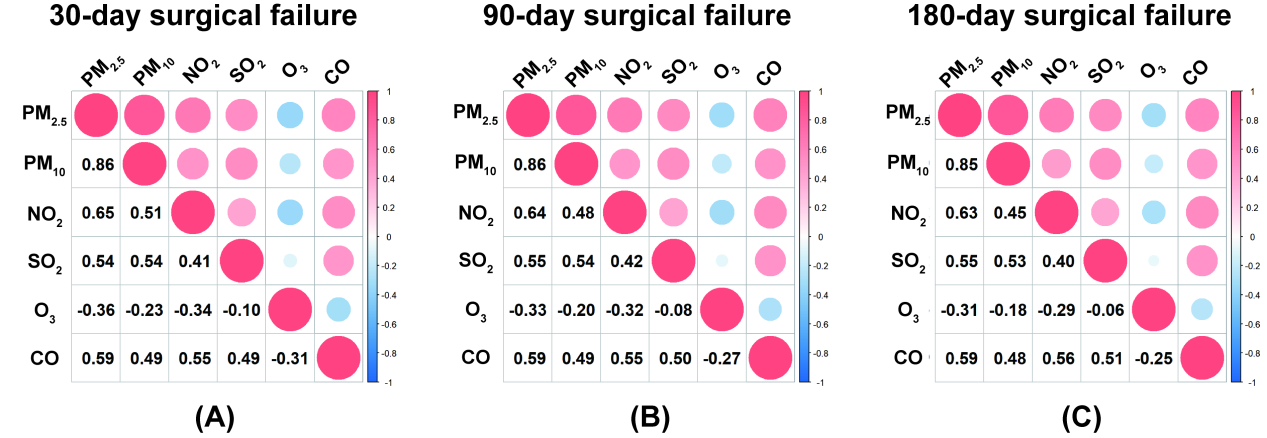


**Figure S9.** **Spearman rank correlation analyses between temperature, PM_2.5_, PM_10_, NO_2_, and CO for (A) 30-, (B) 90- and (C) 180-day suboptimal surgical outcomes.** Temp, temperature; PM_2.5_, particulate matter with an aerodynamic diameter less than 2.5μm; PM_10_, particulate matter with an aerodynamic diameter less than 10μm; NO_2_, nitrogen dioxide; SO_2_, sulfur dioxide; O_3_, ozone; CO, carbon monoxide.

**
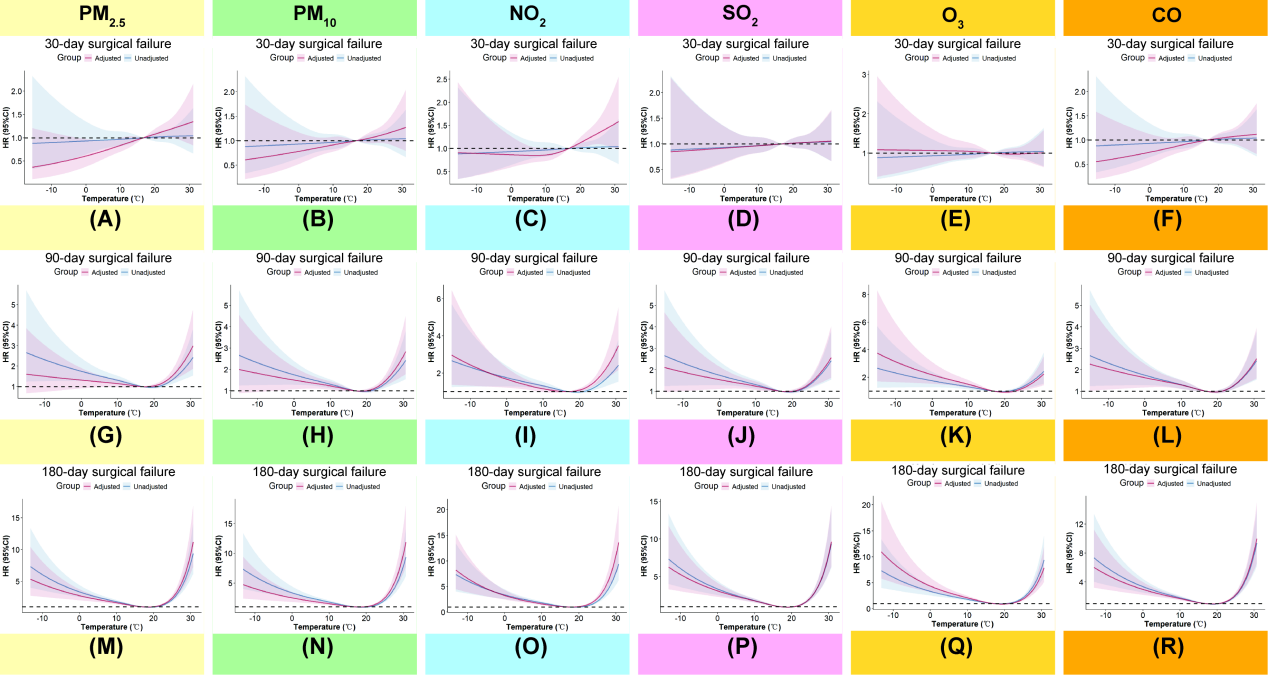
**

**Figure S10.** **RCS curves of different Cox models for ambient temperature at 30, 90, and 180 days post-surgery**. Models of blue curves were adjusted for age, sex, economic geographical location, comorbidities, glaucoma subtype, surgery season, preoperative ophthalmic solutions use, IOP, intraoperative agent and surgery type, while red curves were additionally adjusted for specific air pollutant, including PM_2.5_ ([A], [G] and [M]), PM_10_ ([B], [H] and [N]), NO_2_ ([C], [I] and [O]), SO_2_ ([D], [J] and [P]), O_3_ ([E], [K] and [Q]), and CO ([F], [L] and [R]). RCS, restricted cubic spline; PM_2.5_, particulate matter with an aerodynamic diameter less than 2.5μm; PM_10_, particulate matter with an aerodynamic diameter less than 10μm; NO_2_, nitrogen dioxide; SO_2_, sulfur dioxide; O_3_, ozone; CO, carbon monoxide; IOP, intraocular pressure; HR, hazard ratio; CI, confidence interval.

**Table S1. Categories, generic names, and corresponding brand names of preoperative ophthalmic solutions.**

| **Category** | **Drugs (Generic Name)** | **Brand Names** |
| --- | --- | --- |
| Prostaglandin Analogs | Latanoprost | Xalatan |
|  | Travoprost | Travatan |
|  | Bimatoprost | Lumigan |
|  | Tafluprost | Taflotan |
| β-Blockers | Timolol | Timoptic |
|  | Levobunolol | Betagan |
|  | Carteolol | Mikelan |
|  | Betaxolol | Betoptic |
| α2-Adrenergic Agonists | Brimonidine tartrate | Alphagan |
| Carbonic Anhydrase Inhibitors | Brinzolamide | Azopt |
|  | Dorzolamide | Trusopt |
|  | Acetazolamide | Diamox |
|  | Methazolamide | - |
| Cholinergic Agents | Pilocarpine | - |
| Fixed-combination Preparations | Latanoprost/Timolol | Xalacom |
|  | Travoprost/Timolol | DuoTrav |
|  | Brimonidine/Timolol | Combigan |
|  | Dorzolamide/Timolol | Cosopt |
| Anti-inflammatory | Tobramycin/Dexamethasone | Tobradex |
| ​ | Prednisolone acetate | Pred Forte |
|  | Pranoprofen | Niflan |

**Table S2. Characteristics of participants exposed in lower/higher average ambient temperature 30 days post-surgery.**

| **Characteristic** | **Total (N = 5,193)** | **Q1 (N = 2,596)** | **Q2 (N = 2,597)** | **P value** |
| --- | --- | --- | --- | --- |
| Surgical failure |  |  |  | 0.5793 |
| Yes | 215 (4.1) | 112 (4.3) | 103 (4.0) |  |
| No | 4,978 (95.9) | 2,484 (95.7) | 2,494 (96.0) |  |
| Type of failure |  |  |  | 0.1475 |
| IOP failure | 195 (90.7) | 98 (87.5) | 97 (94.2) |  |
| Reoperation/Revision | 20 (9.3) | 14 (12.5) | 6 (5.8) |  |
| Ambient temperature (°C) |  |  |  | **< 0.0001*** |
| Mean ± SD | 16.22 ± 8.50 | 8.94 ± 5.03 | 23.51 ± 3.61 |  |
| Median (IQR) | 16.64 (9.05-23.70) | 9.05 (5.99-12.71) | 23.70 (20.56-26.45) |  |
| Range | -23.90~33.29 | -23.90~16.64 | 16.64~33.29 |  |
| Age (y) |  |  |  | 0.9023 |
| Mean ± SD | 56.84 ± 14.35 | 56.90 ± 14.28 | 56.78 ± 14.43 |  |
| Median (IQR) | 59.00 (49.00-67.00) | 59.00 (49.00-67.00) | 59.00 (48.00-67.00) |  |
| Range | 18.00~86.00 | 18.00~86.00 | 18.00~86.00 |  |
| Baseline IOP (mmHg) |  |  |  | 0.8936 |
| Mean ± SD | 33.01 ± 11.52 | 32.97 ± 11.23 | 33.05 ± 11.80 |  |
| Median (IQR) | 32.00 (23.00-42.00) | 32.00 (23.77-42.00) | 32.00 (22.60-42.00) |  |
| Range | 15.10~73.00 | 15.10~73.00 | 15.10~72.00 |  |
| Preoperative eye drop duration (m) |  |  |  | 0.1140 |
| Mean ± SD | 10.11 ± 24.81 | 10.75 ± 26.62 | 9.47 ± 22.84 |  |
| Median (IQR) | 3.00 (1.00-11.00) | 3.00 (1.00-12.00) | 3.00 (1.00-9.00) |  |
| Range | 0.00~600.00 | 0.00~600.00 | 0.00~396.00 |  |
| Sex |  |  |  | 0.2129 |
| Male | 2,905 (55.9) | 1,430 (55.1) | 1,475 (56.8) |  |
| Female | 2,288 (44.1) | 1,166 (44.9) | 1,122 (43.2) |  |
| Intraoperative agent |  |  |  | **< 0.0001*** |
| 5-fluorouracil | 1,010 (19.4) | 538 (20.7) | 472 (18.2) |  |
| Mitomycin C | 2,163 (41.7) | 1,142 (44.0) | 1,021 (39.3) |  |
| No drug use | 2,020 (38.9) | 916 (35.3) | 1,104 (42.5) |  |
| Surgery type |  |  |  | 0.6285 |
| Trabeculectomy | 2,583 (49.7) | 1,302 (50.2) | 1,281 (49.3) |  |
| Ex-PRESS shunt | 731 (14.1) | 354 (13.6) | 377 (14.5) |  |
| Ahmed glaucoma valve | 1,879 (36.2) | 940 (36.2) | 939 (36.2) |  |
| Economic geographical location |  |  |  | 0.9832 |
| East | 3,956 (76.2) | 1,972 (76.0) | 1,984 (76.4) |  |
| East-north | 107 (2.1) | 53 (2.0) | 54 (2.1) |  |
| Middle | 993 (19.1) | 502 (19.3) | 491 (18.9) |  |
| West | 137 (2.6) | 69 (2.7) | 68 (2.6) |  |
| Hypertension |  |  |  | **0.0005*** |
| Yes | 1,374 (26.5) | 631 (24.3) | 743 (28.6) |  |
| No | 3,819 (73.5) | 1,965 (75.7) | 1,854 (71.4) |  |
| Diabetes |  |  |  | **0.0060*** |
| Yes | 846 (16.3) | 386 (14.9) | 460 (17.7) |  |
| No | 4,347 (83.7) | 2,210 (85.1) | 2,137 (82.3) |  |
| Glaucoma subtype |  |  |  | **0.0258*** |
| POAG | 1,342 (25.8) | 703 (27.1) | 639 (24.6) |  |
| PACG | 2,641 (50.9) | 1,272 (49.0) | 1,369 (52.7) |  |
| Secondary glaucoma | 1,210 (23.3) | 621 (23.9) | 589 (22.7) |  |
| Preoperative eye drop |  |  |  |  |
| Prostaglandin analogs | 1,753 (33.8) | 945 (36.4) | 808 (31.1) | **< 0.0001*** |
| Beta blockers | 2,177 (41.9) | 1,061 (40.9) | 1,116 (43.0) | 0.1404 |
| Alpha-2 agonists | 2,746 (52.9) | 1,383 (53.3) | 1,363 (52.5) | 0.5693 |
| Carbonic anhydrase inhibitors | 2,321 (44.7) | 1,120 (43.1) | 1,201 (46.2) | **0.0286*** |
| Cholinergic agents | 1,259 (24.2) | 583 (22.5) | 676 (26.0) | **0.0035*** |
| Fixed-combination drugs | 171 (3.3) | 89 (3.4) | 82 (3.2) | 0.6426 |
| Anti-inflammatory drugs | 571 (11.0) | 282 (10.9) | 289 (11.1) | 0.7864 |
| Unknown medications | 388 (7.5) | 185 (7.1) | 203 (7.8) | 0.3675 |
| No medications | 327 (6.3) | 174 (6.7) | 153 (5.9) | 0.2547 |
| Postoperative antifibrotic drugs |  |  |  | 0.5713 |
| Yes | 725 (14.0) | 355 (13.7) | 370 (14.2) |  |
| No | 4,468 (86.0) | 2,241 (86.3) | 2,227 (85.8) |  |
| Surgery season |  |  |  | **< 0.0001*** |
| Spring | 1,587 (30.6) | 537 (20.7) | 1,050 (40.4) |  |
| Summer | 1,196 (23.0) | 8 (0.3) | 1,188 (45.7) |  |
| Autumn | 1,050 (20.2) | 697 (26.8) | 353 (13.6) |  |
| Winter | 1,360 (26.2) | 1,354 (52.2) | 6 (0.2) |  |
| Surgery year |  |  |  | **0.0014*** |
| 2015 | 505 (9.7) | 259 (10.0) | 246 (9.5) |  |
| 2016 | 560 (10.8) | 270 (10.4) | 290 (11.2) |  |
| 2017 | 526 (10.1) | 256 (9.9) | 270 (10.4) |  |
| 2018 | 509 (9.8) | 265 (10.2) | 244 (9.4) |  |
| 2019 | 602 (11.6) | 315 (12.1) | 287 (11.1) |  |
| 2020 | 527 (10.1) | 219 (8.4) | 308 (11.9) |  |
| 2021 | 547 (10.5) | 271 (10.4) | 276 (10.6) |  |
| 2022 | 426 (8.2) | 214 (8.2) | 212 (8.2) |  |
| 2023 | 657 (12.7) | 334 (12.9) | 323 (12.4) |  |
| 2024 | 334 (6.4) | 193 (7.4) | 141 (5.4) |  |

POAG, primary open-angle glaucoma; PACG, primary angle-closure glaucoma; SD: standard deviance; IQR: interquartile range. Bold values highlight the P value of <0.05.

**Table S3. Characteristics of participants exposed in lower/higher average ambient temperature 90 days post-surgery.**

| **Characteristic** | **Total (N = 5,193)** | **Q1 (N = 2,596)** | **Q2 (N = 2,597)** | **P value** |
| --- | --- | --- | --- | --- |
| Suboptimal outcomes |  |  |  | 0.4849 |
| Yes | 583 (11.2) | 300 (11.6) | 283 (10.9) |  |
| No | 4,610 (88.8) | 2,296 (88.4) | 2,314 (89.1) |  |
| Type of failure |  |  |  | **0.0008*** |
| IOP failure | 538 (92.3) | 266 (88.7) | 272 (96.1) |  |
| Reoperation/Revision | 45 (7.7) | 34 (11.3) | 11 (3.9) |  |
| Ambient temperature (°C) |  |  |  | **< 0.0001*** |
| Mean ± SD | 16.44 ± 7.98 | 9.63 ± 4.87 | 23.24 ± 3.32 |  |
| Median (IQR) | 16.89 (9.82-23.66) | 9.82 (6.94-13.26) | 23.66 (20.40-25.82) |  |
| Range | -23.90~31.87 | -23.90~16.89 | 16.89~31.87 |  |
| Age (y) |  |  |  | 0.7421 |
| Mean ± SD | 56.84 ± 14.35 | 56.91 ± 14.41 | 56.78 ± 14.30 |  |
| Median (IQR) | 59.00 (49.00-67.00) | 59.00 (49.00-68.00) | 59.00 (48.00-67.00) |  |
| Range | 18.00~86.00 | 18.00~86.00 | 18.00~86.00 |  |
| Baseline IOP (mmHg) |  |  |  | 0.6375 |
| Mean ± SD | 33.01 ± 11.52 | 33.02 ± 11.25 | 33.00 ± 11.78 |  |
| Median (IQR) | 32.00 (23.00-42.00) | 32.00 (23.60-42.00) | 32.00 (22.80-42.00) |  |
| Range | 15.10~73.00 | 15.10~73.00 | 15.10~72.00 |  |
| Preoperative eye drop duration (m) |  |  |  | 0.2643 |
| Mean ± SD | 200.96 ± 2141.00 | 243.17 ± 2361.35 | 158.76 ± 1894.82 |  |
| Median (IQR) | 3.00 (1.00-11.00) | 3.00 (1.00-12.00) | 3.00 (1.00-9.00) |  |
| Range | 0.00~24276.00 | 0.00~24276.00 | 0.00~24276.00 |  |
| Sex |  |  |  | 0.5102 |
| Male | 2,906 (56.0) | 1,440 (55.5) | 1,466 (56.4) |  |
| Female | 2,287 (44.0) | 1,156 (44.5) | 1,131 (43.6) |  |
| Intraoperative agent |  |  |  | **< 0.0001*** |
| 5-fluorouracil | 1,010 (19.4) | 549 (21.1) | 461 (17.8) |  |
| Mitomycin C | 2,164 (41.7) | 1,128 (43.5) | 1,036 (39.9) |  |
| No drug use | 2,019 (38.9) | 919 (35.4) | 1,100 (42.4) |  |
| Surgery type |  |  |  | 0.2586 |
| Trabeculectomy | 2,583 (49.7) | 1,301 (50.1) | 1,282 (49.4) |  |
| Ex-PRESS shunt | 731 (14.1) | 345 (13.3) | 386 (14.9) |  |
| Ahmed glaucoma valve | 1,879 (36.2) | 950 (36.6) | 929 (35.8) |  |
| Economic geographical location |  |  |  | 0.5948 |
| East | 3,956 (76.2) | 1,968 (75.8) | 1,988 (76.5) |  |
| East-north | 107 (2.1) | 49 (1.9) | 58 (2.2) |  |
| Middle | 993 (19.1) | 506 (19.5) | 487 (18.8) |  |
| West | 137 (2.6) | 73 (2.8) | 64 (2.5) |  |
| Hypertension |  |  |  | **0.0003*** |
| Yes | 1,373 (26.4) | 628 (24.2) | 745 (28.7) |  |
| No | 3,820 (73.6) | 1,968 (75.8) | 1,852 (71.3) |  |
| Diabetes |  |  |  | **0.0419*** |
| Yes | 847 (16.3) | 396 (15.3) | 451 (17.4) |  |
| No | 4,346 (83.7) | 2,200 (84.7) | 2,146 (82.6) |  |
| Glaucoma subtype |  |  |  | **0.0395*** |
| POAG | 1,342 (25.8) | 689 (26.5) | 653 (25.1) |  |
| PACG | 2,642 (50.9) | 1,276 (49.2) | 1,366 (52.6) |  |
| Secondary glaucoma | 1,209 (23.3) | 631 (24.3) | 578 (22.3) |  |
| Preoperative eye drop |  |  |  |  |
| Prostaglandin analogs | 1,753 (33.8) | 920 (35.4) | 833 (32.1) | **0.0101*** |
| Beta blockers | 2,178 (41.9) | 1,054 (40.6) | 1,124 (43.3) | 0.0509 |
| Alpha-2 agonists | 2,747 (52.9) | 1,390 (53.5) | 1,357 (52.3) | 0.3816 |
| Carbonic anhydrase inhibitors | 2,322 (44.7) | 1,127 (43.4) | 1,195 (46.0) | 0.0678 |
| Cholinergic agents | 1,259 (24.2) | 598 (23.0) | 661 (25.5) | **0.0439*** |
| Fixed-combination drugs | 171 (3.3) | 88 (3.4) | 83 (3.2) | 0.7577 |
| Anti-inflammatory drugs | 570 (11.0) | 271 (10.4) | 299 (11.5) | 0.2288 |
| Unknown medications | 388 (7.5) | 191 (7.4) | 197 (7.6) | 0.7888 |
| No medications | 327 (6.3) | 178 (6.9) | 149 (5.7) | 0.1105 |
| Postoperative antifibrotic drugs |  |  |  | 0.5535 |
| Yes | 855 (16.5) | 436 (16.8) | 419 (16.1) |  |
| No | 4,338 (83.5) | 2,160 (83.2) | 2,178 (83.9) |  |
| Surgery season |  |  |  | **< 0.0001*** |
| Spring | 1,587 (30.6) | 395 (15.2) | 1,192 (45.9) |  |
| Summer | 1,195 (23.0) | 49 (1.9) | 1,146 (44.1) |  |
| Autumn | 1,050 (20.2) | 838 (32.3) | 212 (8.2) |  |
| Winter | 1,361 (26.2) | 1,314 (50.6) | 47 (1.8) |  |
| Surgery year |  |  |  | **0.0068*** |
| 2015 | 505 (9.7) | 265 (10.2) | 240 (9.2) |  |
| 2016 | 560 (10.8) | 268 (10.3) | 292 (11.2) |  |
| 2017 | 526 (10.1) | 258 (9.9) | 268 (10.3) |  |
| 2018 | 509 (9.8) | 259 (10.0) | 250 (9.6) |  |
| 2019 | 602 (11.6) | 327 (12.6) | 275 (10.6) |  |
| 2020 | 527 (10.1) | 229 (8.8) | 298 (11.5) |  |
| 2021 | 547 (10.5) | 262 (10.1) | 285 (11.0) |  |
| 2022 | 426 (8.2) | 212 (8.2) | 214 (8.2) |  |
| 2023 | 657 (12.7) | 327 (12.6) | 330 (12.7) |  |
| 2024 | 334 (6.4) | 189 (7.3) | 145 (5.6) |  |

POAG, primary open-angle glaucoma; PACG, primary angle-closure glaucoma; SD: standard deviance; IQR: interquartile range. Bold values highlight the P value of <0.05.

**Table S4. Cox proportional hazard ratios for ambient temperature within various postoperative periods.**

| **Temperature (**°**C)** | **Model 1** | |  | **Model 2** | |  | **Model 3** | |  | **Model 4** | |  |
| --- | --- | --- | --- | --- | --- | --- | --- | --- | --- | --- | --- | --- |
|  | **HR (95%CI)** | **P value** | | **HR (95%CI)** | **P value** | | **HR (95%CI)** | **P value** | | **HR (95%CI)** | **P value** | |
| **30-day suboptimal outcome** |  |  | |  |  | |  |  | |  |  | |
| Continuous variable per 1°C | 1.003 (0.991-1.016) | 0.6362 | | 1.006 (0.992-1.019) | 0.4054 | | 1.006 (0.992-1.019) | 0.4035 | | 0.998 (0.972-1.025) | 0.8928 | |
| Quartile |  |  | |  |  | |  |  | |  |  | |
| Q1 (-23.90 ~ 9.05) | Reference |  | | Reference |  | | Reference |  | | Reference |  | |
| Q2 (9.06 ~ 16.64) | 1.153 (0.860-1.546) | 0.3412 | | 1.262 (0.924-1.723) | 0.1441 | | 1.261 (0.924-1.723) | 0.1442 | | 1.111 (0.753-1.638) | 0.5956 | |
| Q3 (16.64 ~ 23.7) | 1.093 (0.814-1.468) | 0.5524 | | 1.175 (0.859-1.608) | 0.3127 | | 1.175 (0.859-1.608) | 0.3135 | | 0.999 (0.618-1.615) | 0.9975 | |
| Q4 (23.71 ~ 33.29) | 1.117 (0.831-1.502) | 0.4624 | | 1.256 (0.915-1.725) | 0.1587 | | 1.257 (0.915-1.726) | 0.1582 | | 1.178 (0.649-2.137) | 0.5905 | |
| P for trend |  | 0.5654 | |  | 0.2313 | |  | 0.2308 | |  | 0.7383 | |
| **90-day suboptimal outcome** |  |  | |  |  | |  |  | |  |  | |
| Continuous variable per 1°C | 0.994 (0.978-1.010) | 0.4429 | | 0.997 (0.984-1.009) | 0.5857 | | 0.997 (0.984-1.009) | 0.5833 | | 0.980 (0.958-1.003) | 0.0816 | |
| Quartile |  |  | |  |  | |  |  | |  |  | |
| Q1 (-23.90 ~ 9.82) | Reference |  | | Reference |  | | Reference |  | | Reference |  | |
| Q2 (9.83 ~ 16.89) | 0.815 (0.575-1.157) | 0.2535 | | 0.806 (0.620-1.049) | 0.1082 | | 0.807 (0.621-1.049) | 0.1094 | | 0.689 (0.510-0.931) | **0.0152*** | |
| Q3 (16.89 ~ 23.66) | 0.756 (0.532-1.076) | 0.1204 | | 0.813 (0.624-1.059) | 0.1251 | | 0.812 (0.623-1.059) | 0.1240 | | 0.607 (0.398-0.927) | **0.0208*** | |
| Q4 (23.66 ~ 33.29) | 0.884 (0.623-1.255) | 0.4907 | | 0.913 (0.701-1.189) | 0.5000 | | 0.913 (0.701-1.189) | 0.4996 | | 0.621 (0.390-0.991) | **0.0459*** | |
| P for trend |  | 0.4343 | |  | 0.5255 | |  | 0.5231 | |  | **0.0494*** | |
| **180-day suboptimal outcome** |  |  | |  |  | |  |  | |  |  | |
| Continuous variable per 1°C | 0.994 (0.981-1.008) | 0.3947 | | 0.995 (0.982-1.008) | 0.4661 | | 0.995 (0.982-1.009) | 0.4728 | | 0.986 (0.966-1.006) | 0.1778 | |
| Quartile |  |  | |  |  | |  |  | |  |  | |
| Q1 (-23.90 ~ 10.81) | Reference |  | | Reference |  | | Reference |  | | Reference |  | |
| Q2 (10.81 ~ 16.94) | 0.537 (0.415-0.697) | **< 0.0001*** | | 0.475 (0.337-0.669) | **< 0.0001*** | | 0.475 (0.338-0.669) | **< 0.0001*** | | 0.477 (0.350-0.651) | **< 0.0001*** | |
| Q3 (16.94 ~ 22.92) | 0.510 (0.393-0.662) | **< 0.0001*** | | 0.450 (0.319-0.634) | **< 0.0001*** | | 0.450 (0.319-0.634) | **< 0.0001*** | | 0.476 (0.331-0.687) | **< 0.0001*** | |
| Q4 (22.93 ~ 33.29) | 0.937 (0.732-1.200) | 0.6052 | | 0.948 (0.680-1.321) | 0.7510 | | 0.949 (0.681-1.322) | 0.7571 | | 0.776 (0.517-1.163) | 0.2195 | |
| P for trend |  | 0.5380 | |  | 0.6671 | |  | 0.6740 | |  | 0.3903 | |

Model 1: Crude; Model 2: Model 1 additionally adjusted for age, sex, economic geographical location, comorbidities and glaucoma subtype; Model 3: Model 2 additionally adjusted for preoperative ophthalmic solutions use; Model 4: Model 3 additionally adjusted for surgery season, IOP, intraoperative agent and surgery type. IOP, intraocular pressure; HR, hazard ratio; CI, confidence interval; Q, quartile. Bold values highlight the P value of <0.05.

**Table S5. The comparison of linear Cox model and RCS Cox model.**

| **Index** | **30-day surgical failure** | **90-day surgical failure** | **180-day surgical failure** |
| --- | --- | --- | --- |
| **LRT** |  |  |  |
| Linear Cox Loglik | -3921.94 | -4914.07 | -5331.39 |
| RCS COX Loglik | -3921.44 | -4898.3 | -5264.45 |
| χ² | 1.008 | 31.526 | 133.878 |
| P value | 0.799 | **< 0.001*** | **< 0.001*** |
| **AIC/BIC** |  |  |  |
| Linear COX AIC | 7871.88 | 9856.13 | 10690.78 |
| RCS COX AIC | 7874.87 | 9828.6 | 10560.9 |
| ΔAIC | 2.99 | -27.53 | -129.88 |
| Linear COX BIC | 7963.65 | 9947.9 | 10782.55 |
| RCS COX BIC | 7979.75 | 9933.48 | 10665.78 |
| ΔBIC | 16.1 | -14.42 | -116.77 |
| **Internal validation** |  |  |  |
| Optimism (95%CI) | -0.0115 (-0.0322~0.0125) | -0.0086 (-0.0306~0.0116) | -0.0065 (-0.0268~0.0132) |

Loglik, log-likelihood; AIC, Akaike Information Criterion; BIC, Bayesian Information Criterion.

**Table S6. Two segmented random-effects Cox regression on 90-, and 180-day suboptimal surgical outcomes.**

| **Temperature (**°**C)** | **HR (95%CI)** | **P value** |
| --- | --- | --- |
| **90-day suboptimal outcomes** |  |  |
| Temperature < 19.2 °C | 0.942 (0.924-0.960) | **< 0.0001*** |
| Temperature ≥ 19.2 °C | 1.093 (1.040-1.149) | **0.0005*** |
| **180-day suboptimal outcomes** |  |  |
| Temperature < 19.0 °C | 0.924 (0.906-0.942) | **< 0.0001*** |
| Temperature ≥ 19.0 °C | 1.300 (1.241-1.362) | **< 0.0001*** |

The model was adjusted for age, sex, economic geographical location, comorbidities, glaucoma subtype, surgery season, preoperative ophthalmic solutions use, IOP, intraoperative agent and surgery type. The HRs indicates an increased risk for every degree Celsius rise in ambient temperature. 20.2 °C and 19.3 °C respectively corresponds to minimum-risk temperature for 120- and 180-day post-surgery. IOP, intraocular pressure; HR, hazard ratio; CI, confidence interval. Bold values highlight the P value of <0.05.

**Table S7. Inflection points and 95%CIs of ambient temperature on suboptimal surgical outcomes.**

| **Outcome** | **Sample size** | **Inflection point (**°**C)** | **95%CI** |
| --- | --- | --- | --- |
| **90-day suboptimal outcomes** |  |  |  |
| Temperature < 19.2 °C | 2,997 | 2.86 | 2.38-4.45 |
| Temperature ≥ 19.2 °C | 2,196 | 26.11 | 26.61-26.39 |
| **180-day suboptimal outcomes** |  |  |  |
| Temperature < 19.0 °C | 2,971 | 3.47 | 2.87-5.01 |
| Temperature ≥ 19.0 °C | 2,222 | 23.97 | 23.39-24.53 |

Maximum likelihood estimation to determine temperature inflection points and validated findings using bootstrap resampling to establish CIs. CI: confidence interval.

**Table S8. Air pollutant exposure of participants within 30, 90 and 180 days post-surgery.**

| Air pollutant | | 30-day suboptimal outcome | 90-day suboptimal outcome | 180-day suboptimal outcome |
| --- | --- | --- | --- | --- |
| PM_2.5_ (μg/m^3^) | Mean ± SD | 39.32 ± 18.32 | 38.87 ± 17.46 | 38.87 ± 17.46 |
|  | Median (IQR) | 35.79 (25.93-48.85) | 35.79 (26.05-47.85) | 35.79 (26.05-47.85) |
|  | Range | 5.78-185.42 | 5.33-185.42 | 5.33-185.42 |
| PM_10_ (μg/m^3^) | Mean ± SD | 64.01 ± 28.36 | 63.48 ± 27.65 | 63.48 ± 27.65 |
|  | Median (IQR) | 58.70 (43.55-78.59) | 57.96 (43.69-77.19) | 57.96 (43.69-77.19) |
|  | Range | 8.96-332.83 | 8.96-332.83 | 8.96-332.83 |
| NO_2_ (μg/m^3^) | Mean ± SD | 33.61 ± 13.76 | 33.31 ± 13.14 | 33.31 ± 13.14 |
|  | Median (IQR) | 32.36 (23.30-42.80) | 32.44 (23.55-42.03) | 32.44 (23.55-42.03) |
|  | Range | 2.59-97.12 | 2.59-84.89 | 2.59-84.89 |
| SO_2_ (μg/m^3^) | Mean ± SD | 10.83 ± 7.47 | 10.69 ± 7.07 | 10.69 ± 7.07 |
|  | Median (IQR) | 8.53 (6.33-13.14) | 8.50 (6.36-13.10) | 8.50 (6.36-13.10) |
|  | Range | 1.33-109.36 | 1.33-98.87 | 1.33-98.87 |
| O_3_ (μg/m^3^) | Mean ± SD | 98.95 ± 30.64 | 99.32 ± 28.48 | 99.32 ± 28.48 |
|  | Median (IQR) | 100.18 (74.85-121.95) | 100.27 (77.64-120.07) | 100.27 (77.64-120.07) |
|  | Range | 18.09-214.88 | 18.09-214.88 | 18.09-214.88 |
| CO (mg/m^3^) | Mean ± SD | 0.75 ± 0.23 | 0.75 ± 0.22 | 0.75 ± 0.22 |
|  | Median (IQR) | 0.72 (0.61-0.85) | 0.72 (0.61-0.84) | 0.72 (0.61-0.84) |
|  | Range | 0.10-2.69 | 0.11-2.69 | 0.11-2.69 |

PM_2.5_, particulate matter with an aerodynamic diameter less than 2.5μm; PM_10_, particulate matter with an aerodynamic diameter less than 10μm; NO_2_, nitrogen dioxide; SO_2_, sulfur dioxide; O_3_, ozone; CO, carbon monoxide.

**Table S9. Cox proportional hazard ratios for air pollutants.**

| Air pollutant  Continuous variable per unit | 30-day suboptimal outcome | |  | 90-day suboptimal outcome | |  | 180-day suboptimal outcome | |  |
| --- | --- | --- | --- | --- | --- | --- | --- | --- | --- |
|  | HR (95%CI) | P value | | HR (95%CI) | P value | | HR (95%CI) | P value | |
| PM_2.5_ (μg/m^3^) | 1.009 (1.003-1.014) | **0.0014*** | | 1.012 (1.007-1.017) | **< 0.0001*** | | 1.014 (1.009-1.019) | **< 0.0001*** | |
| PM_10_ (μg/m^3^) | 1.004 (1.001-1.007) | **0.0249*** | | 1.005 (1.002-1.008) | **0.0011*** | | 1.007 (1.004-1.010) | **< 0.0001*** | |
| NO_2_ (μg/m^3^) | 1.017 (1.010-1.025) | **< 0.0001*** | | 1.019 (1.012-1.026) | **< 0.0001*** | | 1.018 (1.010-1.025) | **< 0.0001*** | |
| SO_2_ (μg/m^3^) | 1.002 (0.990-1.015) | 0.7441 | | 1.011 (1.001-1.022) | **0.0393*** | | 1.016 (1.005-1.027) | **0.0030*** | |
| O_3_ (μg/m^3^) | 1.002 (0.998-1.006) | 0.4275 | | 1.000 (0.997-1.004) | 0.8542 | | 1.002 (0.998-1.005) | 0.4187 | |
| CO (mg/m^3^) | 1.886 (1.270-2.801) | **0.0017*** | | 2.007 (1.390-2.898) | **0.0002*** | | 2.275 (1.580-3.276) | **< 0.0001*** | |

The model was adjusted for age, sex, economic geographical location, comorbidities, glaucoma subtype, surgery season, preoperative ophthalmic solutions use, IOP, intraoperative agent and surgery type. The HRs indicates an increased risk for 1 unit rise in air pollutant. PM_2.5_, particulate matter with an aerodynamic diameter less than 2.5μm; PM_10_, particulate matter with an aerodynamic diameter less than 10μm; NO_2_, nitrogen dioxide; SO_2_, sulfur dioxide; O_3_, ozone; CO, carbon monoxide; HR, hazard ratio; CI, confidence interval. Bold values highlight the P value of <0.05.

**Table S10. Interaction analyses with the additive interaction analyses using MOVER method.**

| Outcomes and adjusted pollutant | Multiplicative scale | P for multiplicative interaction | RERI (95%CI) | AP (95%CI) | SI (95%CI) |
| --- | --- | --- | --- | --- | --- |
| **30-day suboptimal outcome** |  |  |  |  |  |
| PM_2.5_ (μg/m^3^) | 0.98 (0.95-1.01) | 0.1808 | -0.01 (-0.70~0.63) | -0.01 (-0.16~0.17) | 0.99 (0.96-1.02) |
| PM_10_ (μg/m^3^) | 0.99 (0.97-1.02) | 0.6459 | -0.00 (-0.34~0.29) | -0.00 (-0.16~0.19) | 0.99 (0.93-1.05) |
| NO_2_ (μg/m^3^) | 1.00 (0.97-1.02) | 0.7960 | 0.00 (-0.32~0.28) | 0.00 (-0.14~0.17) | 1.00 (0.93-1.07) |
| SO_2_ (μg/m^3^) | 1.00 (0.98-1.02) | 0.9202 | 0.00 (-0.25~0.22) | 0.00 (-0.12~0.16) | 0.01 (0.92-1.11) |
| O_3_ (μg/m^3^) | 0.99 (0.96-1.02) | 0.3800 | -0.02 (-0.48~0.36) | -0.01 (-0.19~0.22) | **0.96 (0.92-1.00)** |
| CO (mg/m^3^) | 0.99 (0.97-1.01) | 0.3459 | -0.01 (-0.29~0.22) | -0.01 (-0.16~0.17) | 0.95 (0.89-1.01) |
| **90-day suboptimal outcome** |  |  |  |  |  |
| PM_2.5_ (μg/m^3^) | 0.96 (0.93-0.98) | **0.0013*** | -0.07 (-0.91~0.66) | -0.03 (-0.13~0.09) | **0.96 (0.94-0.98)** |
| PM_10_ (μg/m^3^) | 0.97 (0.94-0.99) | **0.0105*** | -0.05 (-0.63~0.45) | -0.02 (-0.11~0.09) | **0.96 (0.94-0.98)** |
| NO_2_ (μg/m^3^) | 0.97 (0.94-0.99) | **0.0060*** | -0.05 (-0.50~0.31) | -0.03 (-0.11~0.08) | **0.95 (0.93-0.97)** |
| SO_2_ (μg/m^3^) | 0.98 (0.96-1.01) | 0.1566 | -0.02 (-0.37~0.26) | -0.01 (-0.09~0.09) | **0.97 (0.95-1.00)** |
| O_3_ (μg/m^3^) | 1.02 (0.99-1.05) | 0.1263 | 0.02 (-0.23~0.23) | 0.03 (-0.26~0.40) | NA |
| CO (mg/m^3^) | 0.97 (0.95-0.99) | **0.0124*** | -0.04 (-0.37~0.22) | -0.03 (-0.12~0.10) | 0.94 (0.91-0.97) |
| **180-day suboptimal outcome** |  |  |  |  |  |
| PM_2.5_ (μg/m^3^) | 0.90 (0.87-0.93) | **< 0.0001*** | -0.35 (-3.86~2.65) | -0.04 (-0.09~0.01) | **0.95 (0.94-0.97)** |
| PM_10_ (μg/m^3^) | 0.92 (0.90-0.95) | **< 0.0001*** | -0.20 (-1.96~1.28) | -0.04 (-0.09~0.02) | **0.96 (0.94-0.97)** |
| NO_2_ (μg/m^3^) | 0.93 (0.90-0.95) | **< 0.0001*** | -0.19 (-1.18~0.56) | -0.05 (-0.10~0.02) | **0.94 (0.92-0.95)** |
| SO_2_ (μg/m^3^) | 0.96 (0.94-0.99) | **0.0017*** | -0.07 (-0.65~0.41) | -0.03 (-0.09~0.06) | **0.96 (0.94-0.98)** |
| O_3_ (μg/m^3^) | 1.08 (1.05-1.11) | **< 0.0001*** | 0.06 (-0.08~0.17) | 0.15 (-0.54~1.08) | NA |
| CO (mg/m^3^) | 0.94 (0.92-0.96) | **< 0.0001*** | -0.12 (-0.80~0.40) | -0.04 (-0.11~0.04) | **0.93 (0.92-0.95)** |

The model was adjusted for specific air pollutant, age, sex, economic geographical location, comorbidities, glaucoma subtype, surgery season, preoperative ophthalmic solutions use, IOP, intraoperative agent and surgery type. In the model, air pollutant was considered as a binary variable. Both multiplicative and additive interaction analyses were performed. “MOVER” method was selected to conduct additive interaction analyses. PM_2.5_, particulate matter with an aerodynamic diameter less than 2.5μm; PM_10_, particulate matter with an aerodynamic diameter less than 10μm; NO_2_, nitrogen dioxide; SO_2_, sulfur dioxide; O_3_, ozone; CO, carbon monoxide; RERI, relative excess risks due to interaction; AP, attributable proportion due to interaction; SI, synergy index; IOP, intraocular pressure; CI, confidence interval. Bold values highlight the P value of <0.05, RERI < 0, AP < 0 or SI < 1.

**Table S11. Sensitivity analysis on interaction effects between ambient temperature and air pollutants using simple asymptotic method.**

| Outcomes and adjusted pollutant | Multiplicative scale | P for multiplicative interaction | RERI (95%CI) | AP (95%CI) | SI (95%CI) |
| --- | --- | --- | --- | --- | --- |
| **30-day suboptimal outcome** |  |  |  |  |  |
| PM_2.5_ (μg/m^3^) | 0.81 (0.52-1.25) | 0.3348 | -0.18 (-0.77~0.42) | -0.09 (-0.35~0.42) | 0.85 (0.57-1.28) |
| PM_10_ (μg/m^3^) | 1.01 (0.68-1.50) | 0.9600 | 0.03 (-0.36~0.41) | 0.02 (-0.29~0.33) | 1.11 (0.18-6.82) |
| NO_2_ (μg/m^3^) | 0.98 (0.66-1.46) | 0.9288 | 0.02 (-0.38~0.42) | 0.01 (-0.27~0.29) | 1.04 (0.40-2.74) |
| SO_2_ (μg/m^3^) | 1.36 (0.95-1.95) | 0.0951 | 0.28 (0.05-0.52) | 0.28 (-0.06~0.62) | NA |
| O_3_ (μg/m^3^) | 0.84 (0.54-1.31) | 0.4405 | -0.21 (-0.83~0.40) | -0.16 (-0.55~0.23) | 0.61 (0.35-1.06) |
| CO (mg/m^3^) | 0.96 (0.66-1.40) | 0.8426 | -0.04 (-0.42~0.34) | -0.04 (-0.37~0.30) | 0.71 (0.10-4.99) |
| **90-day suboptimal outcome** |  |  |  |  |  |
| PM_2.5_ (μg/m^3^) | 0.71 (0.49-1.04) | 0.0814 | -0.48 (-1.18~0.22) | -0.28 (-0.60~0.03) | **0.59 (0.39-0.90)** |
| PM_10_ (μg/m^3^) | 0.91 (0.64-1.29) | 0.5972 | -0.11 (-0.56~0.35) | -0.07 (-0.36~0.22) | 0.81 (0.42-1.54) |
| NO_2_ (μg/m^3^) | 0.78 (0.55-1.11) | 0.1654 | -0.33 (-0.89~0.23) | -0.22 (-0.52~0.08) | **0.60 (0.38-0.95)** |
| SO_2_ (μg/m^3^) | 1.06 (0.77-1.46) | 0.7371 | 0.05 (-0.30~0.41) | 0.04 (-0.25~0.33) | 1.22 (0.20-7.32) |
| O_3_ (μg/m^3^) | 0.94 (0.63-1.40) | 0.7716 | -0.09 (-0.59~0.41) | -0.09 (-0.53~0.35) | 0.34 (0.01-11.57) |
| CO (mg/m^3^) | 0.79 (0.57-1.11) | 0.1722 | -0.29 (-0.78~0.20) | -0.25 (-0.59~0.10) | 0.36 (0.09-1.38) |
| **180-day suboptimal outcome** |  |  |  |  |  |
| PM_2.5_ (μg/m^3^) | 0.65 (0.45-0.94) | **0.0214*** | -0.66 (-1.48~0.16) | **-0.30 (-0.58~-0.02)** | **0.65 (0.48-0.88)** |
| PM_10_ (μg/m^3^) | 0.72 (0.51-1.01) | 0.0552 | -0.47 (-1.13~0.18) | -0.24 (-0.50~0.02) | **0.68 (0.50-0.93)** |
| NO_2_ (μg/m^3^) | 0.66 (0.47-0.92) | **0.0146*** | -0.63 (-1.31~0.06) | **-0.38 (-0.68~-0.07)** | **0.51 (0.35-0.74)** |
| SO_2_ (μg/m^3^) | 0.91 (0.67-1.24) | 0.5440 | -0.13 (-0.57~0.31) | -0.09 (-0.34~0.17） | 0.80 (0.49-1.33) |
| O_3_ (μg/m^3^) | 1.13 (0.77-1.65) | 0.5348 | 0.09 (-0.31~0.49) | 0.10 (-0.37~0.57) | NA |
| CO (mg/m^3^) | 0.68 (0.49-0.94) | **0.0194*** | -0.54 (-1.14~0.06) | **-0.39 (-0.70~-0.07)** | **0.42 (0.25-0.72)** |

The model was adjusted for specific air pollutant, age, sex, economic geographical location, comorbidities, glaucoma subtype, surgery season, preoperative ophthalmic solutions use, IOP, intraoperative agent and surgery type. In the model, air pollutant and ambient temperature were both considered as binary variables. Both multiplicative and additive interaction analyses were performed. Simple asymptotic method was selected to conduct additive interaction analyses. PM_2.5_, particulate matter with an aerodynamic diameter less than 2.5μm; PM_10_, particulate matter with an aerodynamic diameter less than 10μm; NO_2_, nitrogen dioxide; SO_2_, sulfur dioxide; O_3_, ozone; CO, carbon monoxide; POAG, primary open-angle glaucoma; PACG, primary angle-closure glaucoma; RERI, relative excess risks due to interaction; AP, attributable proportion due to interaction; SI, synergy index; IOP, intraocular pressure; CI, confidence interval. Bold values highlight the P value of <0.05, RERI < 0, AP < 0 or SI < 1.

**Table S12. Sensitivity analysis on interaction effects between ambient temperature and air pollutants using MOVER method.**

| Outcomes and adjusted pollutant | Multiplicative scale | P for multiplicative interaction | RERI (95%CI) | AP (95%CI) | SI (95%CI) |
| --- | --- | --- | --- | --- | --- |
| **30-day suboptimal outcome** |  |  |  |  |  |
| PM_2.5_ (μg/m^3^) | 0.81 (0.52-1.25) | 0.3348 | -0.18 (-1.77~0.55) | -0.09 (-0.35~0.27) | 0.85 (0.57-1.28) |
| PM_10_ (μg/m^3^) | 1.01 (0.68-1.50) | 0.9600 | 0.03 (-0.83~0.30) | 0.02 (-0.24~0.47) | 1.11 (0.18-6.82) |
| NO_2_ (μg/m^3^) | 0.98 (0.66-1.46) | 0.9288 | 0.01 (-0.90~0.36) | 0.01 (-0.23~0.40) | 1.04 (0.40-2.74) |
| SO_2_ (μg/m^3^) | 1.36 (0.95-1.95) | 0.0951 | 0.28 (-0.22~0.45) | 0.28 (0.02-0.75) | NA |
| O_3_ (μg/m^3^) | 0.84 (0.54-1.31) | 0.4405 | **-0.21 (-1.42~-0.01)** | -0.16 (-0.51~0.32) | 0.61 (0.35-1.06) |
| CO (mg/m^3^) | 0.96 (0.66-1.40) | 0.8426 | -0.04 (-0.78~0.15) | -0.04 (-0.32~0.42) | 0.71 (0.10-4.99) |
| **90-day suboptimal outcome** |  |  |  |  |  |
| PM_2.5_ (μg/m^3^) | 0.71 (0.49-1.04) | 0.0814 | **-0.48 (-1.87~-0.11)** | -0.28 (-0.60~0.09) | **0.59 (0.39-0.90)** |
| PM_10_ (μg/m^3^) | 0.91 (0.64-1.29) | 0.5972 | -0.11 (-0.99~0.15) | -0.07 (-0.34~0.29) | 0.81 (0.42-1.54) |
| NO_2_ (μg/m^3^) | 0.78 (0.55-1.11) | 0.1654 | **-0.33 (-1.36~-0.04)** | -0.22 (-0.51~0.14) | **0.60 (0.38-0.95)** |
| SO_2_ (μg/m^3^) | 1.06 (0.77-1.46) | 0.7371 | 0.05 (-0.60~0.25) | 0.04 (-0.21~0.40) | 1.22 (0.20-7.32) |
| O_3_ (μg/m^3^) | 0.94 (0.63-1.40) | 0.7716 | -0.09 (-0.98~0.11) | -0.09 (-0.48~0.45) | 0.34 (0.01-11.57) |
| CO (mg/m^3^) | 0.79 (0.57-1.11) | 0.1722 | **-0.29 (-1.11~-0.04)** | -0.25 (-0.57~0.16) | 0.36 (0.09-1.38) |
| **180-day suboptimal outcome** |  |  |  |  |  |
| PM_2.5_ (μg/m^3^) | 0.64 (0.45-0.94) | **0.0214*** | **-0.66 (-2.32~-0.18)** | **-0.30 (-0.59~0.00)** | **0.65 (0.48-0.88)** |
| PM_10_ (μg/m^3^) | 0.72 (0.51-1.01) | 0.0552 | **-0.47 (-1.72~-0.11)** | -0.24 (-0.50~0.05) | **0.68 (0.50-0.93)** |
| NO_2_ (μg/m^3^) | 0.66 (0.47-0.92) | **0.0146*** | **-0.63 (-1.81~-0.28)** | **-0.38 (-0.68~-0.04)** | **0.51 (0.35-0.74)** |
| SO_2_ (μg/m^3^) | 0.91 (0.67-1.24) | 0.5440 | -0.13 (-0.92~0.10) | -0.09 (-0.33~0.22) | 0.80 (0.49-1.33) |
| O_3_ (μg/m^3^) | 1.13 (0.77-1.65) | 0.5348 | 0.09 (-0.61~0.26) | 0.10 (-0.30~0.67) | NA |
| CO (mg/m^3^) | 0.68 (0.49-0.94) | **0.0194*** | **-0.54 (-1.53~-0.22)** | **-0.39 (-0.70~-0.03)** | **0.42 (0.25-0.72)** |

The model was adjusted for specific air pollutant, age, sex, economic geographical location, comorbidities, glaucoma subtype, surgery season, preoperative ophthalmic solutions use, IOP, intraoperative agent and surgery type. In the model, air pollutant and ambient temperature were both considered as binary variables. Both multiplicative and additive interaction analyses were performed. MOVER method was selected to conduct additive interaction analyses. PM_2.5_, particulate matter with an aerodynamic diameter less than 2.5μm; PM_10_, particulate matter with an aerodynamic diameter less than 10μm; NO_2_, nitrogen dioxide; SO_2_, sulfur dioxide; O_3_, ozone; CO, carbon monoxide; POAG, primary open-angle glaucoma; PACG, primary angle-closure glaucoma; RERI, relative excess risks due to interaction; AP, attributable proportion due to interaction; SI, synergy index; IOP, intraocular pressure; CI, confidence interval. Bold values highlight the P value of <0.05, RERI < 0, AP < 0 or SI < 1.
